# Integrating genomics and targeted metabolite profiling to elucidate disease-suppression mechanisms of *Bacillus velezensis* GFB08

**DOI:** 10.1016/j.crmicr.2025.100503

**Published:** 2025-10-30

**Authors:** Yi-Tun Cho, Hieng-Ming Ting, Bo-Wei Wang, Yi-Chen Tsai, Hao-Yung Wang, Yu-Liang Yang, Hiran A. Ariyawansa

**Affiliations:** a Department of Plant Pathology and Microbiology, National Taiwan University, Taiwan; b Institute of Plant Biology, National Taiwan University, Taiwan; c Department of Life Science, National Taiwan University, Taiwan; d Agricultural Biotechnology Research Center, Academia Sinica, Taiwan; e Hualien District Agricultural Research and Extension Station, Ministry of Agriculture, Taiwan

**Keywords:** Biocontrol agent, Bioactive secondary metabolites, Comparative genomic analysis, Plant growth promotion, Fermentation optimization

## Abstract

Welsh onion cultivation in Taiwan faces a significant threat from a foliar disease complex caused by three major pathogens: *Stemphylium vesicarium, Colletotrichum spaethianum*, and *C. circinans*. As an alternative to conventional fungicides, the biocontrol agent *Bacillus velezensis* GFB08 was systematically characterised through an integrated genomic and targeted metabolite profiling approach. The 3.89 Mbp genome harbored biosynthetic gene clusters for key lipopeptides, and comparative genomics revealed a mersacidin-like BGC, a feature variably present in B. velezensis, indicating enhanced antimicrobial potential. Metabolite profiling identified fengycin and bacillomycin D as the primary antifungal compounds. Bioassays demonstrated that purified fengycin exhibited potent, broad-spectrum activity against all three pathogens, while bacillomycin D displayed species-specific effects, significantly inhibiting only *C. spaethianum*. The interaction between these two lipopeptides was determined to be additive, not synergistic, against *C. spaethianum*. Furthermore, volatile organic compounds (VOCs) from GFB08 significantly inhibited *C. spaethianum* while unexpectedly stimulating the growth of *C. circinans*, highlighting the complexity of its biocontrol mechanisms. Among these VOCs, acetic acid was highly effective, providing complete inhibition at a concentration of 0.1 µL/mL. The strain also demonstrated a significant plant growth-promoting effect, which was limited to the seed stage. This study provides the first integrative genomic–metabolite–functional characterization of a Welsh onion biocontrol strain GFB08, clarifying its multi-modal mechanisms and highlighting the role of mersacidin-associated genomic features and pathogen-specific VOC responses in biocontrol efficacy.

## Graphical abstract

**Figure:**
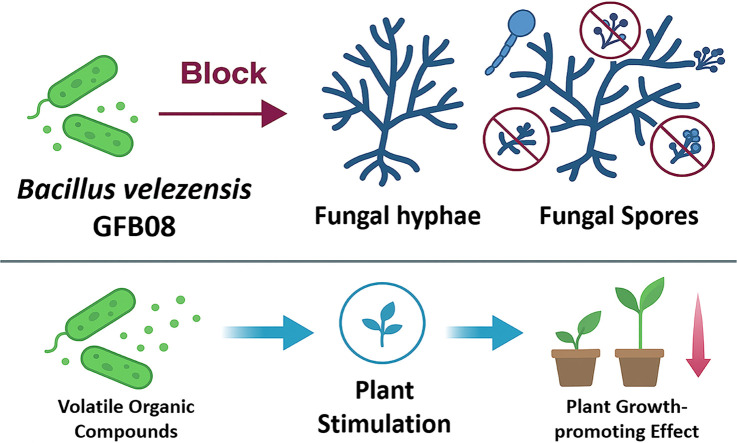

## 1 Introduction

Welsh onion (*Allium fistulosum*) is widely cultivated in Taiwan and is valued for enhancing the flavor of various dishes and cuisines. The main production areas in Taiwan include Yilan, Changhua, and Yunlin, with an annual yield reaching approximately 87,464 tons (Agriculture and Food Agency, 2023). In recent years, foliar diseases have posed a significant threat to Welsh onion production (Tzean et al., 2019). Newly emerging diseases, such as Stemphylium leaf blight (SLB) caused by *Stemphylium vesicarium* and anthracnose caused by *Colletotrichum spaethianum* and *C. circinans*, have frequently been reported to cause outbreaks in Yilan, resulting in up to 10 % yield losses (Wang et al., 2021; Yu et al., 2024). Even though these pathogens are capable of independently causing similar leaf blight symptoms, they exhibit seasonal variations in their occurrence and incidence. SLB is primarily observed in winter and spring, whereas anthracnose occurs year-round (Wang et al., 2021; Yu et al., 2024). These observations suggest that foliar diseases of Welsh onion constitute a disease complex involving multiple pathogens, leading to challenges in disease management (Jayasinghe et al., 2024).

Welsh onion growers in Taiwan heavily rely on fungicide applications to manage foliar diseases. However, the foliar disease of Welsh onion is a complex disease caused by three fungal species, namely *Stemphylium vesicarium, Colletotrichum spaethianum*, and *C. circinans*, in Taiwan (Jayasinghe et al., 2024). Recent studies have demonstrated that these pathogens exhibit varying sensitivities to commonly used fungicides, rendering effective disease management challenging (Yu et al., 2024). These studies reported that among 31 fungicides tested, only difenoconazole, prochloraz, and the combination of cyprodinil and fludioxonil were effective against all three pathogens (Wang et al., 2021; Yu et al., 2024). Variation in fungicide sensitivity among these pathogens has led to excessive fungicide applications, increasing the risk of pesticide residues and environmental contamination (Pimentão et al., 2024). This situation highlights the urgent need for alternative disease management strategies. Biological control agents (BCAs) present a promising solution to these challenges. In recent years, we isolated and screened microbial strains from healthy Welsh onion leaves to identify potential biological control agents against foliar pathogens. Among 109 bacterial and 31 fungal isolates, *Bacillus velezensis* strain GFB08 demonstrated the highest biocontrol efficacy against the foliar disease complex (Wang et al., 2023).

*Bacillus velezensis* is a gram-positive, aerobic bacterium, commonly isolated from soil, roots, and leaves (Serrão et al., 2024). Phylogenetic analyses have shown that it is closely related to *B. amyloliquefaciens* and *B. subtilis* (Dunlap et al., 2016). Its ability to form endospores allows *B. velezensis* to survive in diverse and harsh environments (Karačić et al., 2024), offering a significant advantage when applied as a biological control agent or plant growth-promoting rhizobacterium (PGPR). Moreover, *B. velezensis* produces a variety of secondary metabolites, including cyclic lipopeptides and volatile organic compounds (VOCs), that contribute to its antimicrobial activity and plant growth-promoting capabilities (Wang et al., 2024; Kenfaoui et al., 2024).

Secondary cyclic lipopeptides, including members of the surfactin, fengycin, and iturin families, have been reported for *B. velezensis* and are known to exhibit potent antifungal activity against a wide range of phytopathogens. For example, Yu et al. (2023) reported that fengycin and iturin produced by *B. velezensis* DMW1 inhibited the growth of *Sclerotinia sclerotiorum* and *Rhizoctonia solani*. These lipopeptides are commonly made as mixtures of homologues and isoforms, which vary in fatty acid chain length and composition, with such structural differences significantly affecting their biological activity (). Surfactin acts as a potent biosurfactant with broad-spectrum activity, including antibacterial, antiviral, and antifungal effects. In contrast, iturin (including bacillomycin) and fengycin are mainly responsible for vigorous antifungal activity (Wang et al., 2024). The co-production of multiple lipopeptides has also been shown to produce synergistic effects, thus enhancing overall antimicrobial effectiveness (Ito et al., 2025).

Other than lipopeptides, *B. velezensis* also produces various volatile organic compounds (VOCs) to inhibit fungal growth. VOCs have recently gained attention due to their high diffusion efficiency, strong permeability, and low residual toxicity. Ling et al. (2022) reported that VOCs produced by *B. velezensis* L1 inhibited mycelial growth and conidial germination of *Alternaria iridiaustralis*, resulting in mycelial abnormalities such as twisted and flattened hyphae. These antifungal VOCs can be broadly classified into five classes: alcohols, organic acids, ketones, benzothiazoles, and sulfur-containing compounds (Grahovac et al., 2023). Notably, compounds such as 3-methyl-1-butanol, acetic acid, and acetoin are commonly released by *B. velezensis* and have been linked with strong antifungal activity (Song et al., 2022; Yuan et al., 2025). Furthermore, specific VOCs of *B. velezensis*, such as acetoin, decanes, benzaldehyde, and dimethyl disulfide, are recognized for their ability to promote plant growth (Dutta et al., 2025).

The significant threat posed by foliar diseases to Welsh onion production, combined with the limitations of conventional chemical controls, underscores the urgent need for effective biological alternatives. Although *Bacillus velezensis* GFB08 has demonstrated strong biocontrol potential (Wang et al., 2023), a comprehensive understanding of the disease-suppression mechanisms against the relevant pathogen complex is still lacking. This knowledge gap limits the ability to optimize its application and ensure full consistency in efficacy. Therefore, the main objectives of this study were to: (1) sequence and characterize the genome of *B. velezensis* GFB08, classify the strain using whole-genome and phylogenetic analyses, and perform comparative genomics with reference strains, (2) assess genes and gene clusters related to secondary metabolite production and elucidate their roles in biocontrol activity and plant growth promotion and (3) to identify bioactive secondary metabolites produced by strain GFB08 using Liquid Chromatography-Mass Spectrometry (LC-MS) and Gas Chromatography-Mass Spectrometry (GC–MS).

## 2 Materials and methods

### 2.1 Bacterial strain, media, and genomic DNA extraction

*Bacillus velezensis* GFB08 was cultured in Luria–Bertani (LB) liquid medium at 28 °C with shaking at 150 rpm. Genomic DNA was extracted from a 30 mL overnight culture using a modified cetyltrimethylammonium bromide (CTAB) protocol (Doyle and Doyle, 1987). Briefly, the cell pellet was lysed using 10 % SDS and proteinase K (20 mg/mL). The lysate was purified sequentially with 5 M NaCl, chloroform: isoamyl alcohol (24:1), and phenol: chloroform: isoamyl alcohol (25:24:1). Nucleic acids were precipitated with isopropanol, and the resulting DNA pellet was washed with 80 % ethanol, air-dried, and dissolved in ddH₂O. DNA quality and concentration were assessed using a NanoDrop 1000 spectrophotometer (Thermo Scientific, USA).

### 2.2 Whole-Genome sequencing, assembly, and annotation

The genome of *B. velezensis* GFB08 was sequenced using a hybrid approach combining the Illumina NovaSeq X Plus platform (BIOTOOLS Co., Ltd.) and the Oxford Nanopore GridION platform (NGS High Throughput Genomics Core, Academia Sinica). Illumina raw reads were quality-trimmed using Fastp v0.23.4 (Chen et al., 2018), while Nanopore raw reads were adapter-trimmed using Porechop v0.2.4 (https://github.com/rrwick/Porechop) and further processed with Filtlong v0.2.1 (https://github.com/rrwick/Filtlong). Hybrid genome assembly was implemented using Unicycler v0.5.0 (Wick et al., 2017). Gene annotation was done with the NCBI Prokaryotic Genome Annotation Pipeline (PGAP) (Tatusova et al., 2016). Biosynthetic gene clusters (BGCs) for secondary metabolites were predicted using antiSMASH v7.0 (Blin et al., 2023). A circular genome map was generated using the BLAST Ring Image Generator (BRIG) v0.95 (Alikhan et al., 2011). The average nucleotide identity (ANI) was calculated using pyani v0.2.13.1 (Pritchard et al., 2016). The GFB08 genome was compared with 15 other *Bacillus* genomes (Supplementary Table S1). A pangenome analysis was conducted using anvi'o v8 (Eren et al., 2021) following the official pangenomics workflow. To compare BGCs, a modified version of antiSMASH was used to separate the fengycin and bacillomycin clusters (Steinke et al., 2021). BGC synteny was visualized using Clinker v0.0.31 (Gilchrist and Chooi, 2021). The draft genome of *B. velezensis* GFB08 has been deposited in NCBI under BioProject ID PRJNA1283916, BioSample ID SAMN49705414, and accession number CP195535.

### 2.3 Isolation and identification of bioactive lipopeptides

*B. velezensis* GFB08 was inoculated into 150 mL of LB medium and cultured for 72 h at 28 °C with shaking at 150 rpm. The culture was centrifuged at 16,000 × *g* for 15 min, and the cell-free supernatant was then filtered through a 0.45 μm membrane. Various methods were employed to extract bioactive lipopeptides from the cell-free supernatant. For HP20 adsorption, 2 % (w/v) Diaion HP20 resin was added to the supernatant and incubated overnight at 4 °C. The resin was collected using a sieve and nonwoven fabric, then extracted three times with a minimal volume of methanol under stirring for 30 min. Methanol extracts were pooled and concentrated to dryness using a rotary vacuum evaporator. For ethyl acetate (EA) extraction, the supernatant was partitioned three times with an equal volume of ethyl acetate, and the combined organic phases were evaporated to dryness using a rotary evaporator. For ammonium sulfate precipitation, solid ammonium sulfate was added to the supernatant to achieve 80 % saturation, followed by overnight incubation at 4 °C. The resulting precipitate was recovered by centrifugation at 16,000 × *g* for 15 min at 4 °C and dried using a SpeedVac vacuum concentrator (Thermo Scientific, USA). For acid precipitation, the pH of the supernatant was adjusted to 2.0 with 6 M HCl and was kept overnight at 4 °C. The precipitate was collected by centrifugation at 16,000 × *g* for 15 min at 4 °C, redissolved in methanol, adjusted to neutral pH (7.0) using 2 M NaOH, and dried using a SpeedVac vacuum concentrator (Thermo Scientific, USA).

Purification of fengycin and bacillomycin D from the acid precipitation extract was achieved via two rounds of Sephadex LH-20 column chromatography (4 × 50 cm). The column was eluted isocratically with methanol, and fractions were collected every 15 min. Collected fractions were analyzed by MALDI-TOF-MS (Bruker Daltonics, Germany). For MALDI-TOF-MS analysis, 0.5 μL of the fractions were mixed with 2 μL of a saturated universal MALDI matrix solution (Fluka, St. Gallen, Switzerland) arranged in 50 % acetonitrile containing 0.1 % trifluoroacetic acid (TFA; Sigma, USA). The mixtures were spotted onto a ground steel target plate (MTP 384; Bruker), and the mass spectra were acquired in positive ion and reflection modes with a laser intensity of 40 %.

The chemical profiles of all crude extracts and purified compounds were analyzed on an Agilent 6546 LC/Q-TOF system with an AJS-ESI source. Separation was performed on a Waters ACQUITY UPLC® BEH C18 column (2.1 × 100 mm, 1.7 µm) with a mobile phase of 0.1 % formic acid in water (A) and acetonitrile (B). The gradient was: 5 % B (0–0.5 min); 5–50 % B (0.5–5.8 min); 50–100 % B (5.8–14.5 min); 100 % B (14.5–17.5 min); and 100–5 % B (17.5–17.6 min) at a flow rate of 0.4 mL/min.

### 2.4 Antifungal bioactivity assays of lipopeptides

To evaluate the bioactivity of the lipopeptide extracts obtained using the methods described above, the extracts were resuspended in methanol, diluted to the desired concentrations, and added to 55 mm Petri dishes containing 4 mL PDA. For both cell-free supernatant and crude extracts, the concentrations tested were 125, 250, 500, 1000, and 2000 mg/L. For purified fengycin and bacillomycin D, the concentrations tested were 6.25, 12.5, 25, 50, 100 mg/L. PDA supplemented with 1 % methanol was used as a negative control. Hyphal disks (4 mm diameter) of *Colletotrichum spaethianum* CO15–2, *C. circinans* 30–1, and *Stemphylium vesicarium* SV02 were placed in the center of the PDA plates. All the experiments were conducted with five biological replicates. Colony diameters were measured after four days for *C. spaethianum* CO15–2 and after seven days for *C. circinans* 30–1 and *S. vesicarium* SV02, depending on their mycelial growth rates. The inhibition rate of mycelium growth was calculated as described by Wang et al. (2021):

$$\text{Inhibition}\;\text{rate}\;(\%)\;=\;\frac{\left(\text{Control}\;\text{colony}\;\text{diameter}\;--\;\text{Treatment}\;\text{colony}\;\text{diameter}\right)}{\left(\text{Control}\;\text{colony}\;\text{diameter}\right)}\;\times \;100\;(\%)$$

The IC_50_ values were calculated based on dose–response curves and adapted from methods described in previous studies, using SPSS 240 software version 27.0 (Altın et al., 2018; Yu et al., 2024). Morphological changes of the fungal hyphae treated with purified fengycin and bacillomycin D were then observed and imaged using a Hitachi TM3000 tabletop scanning electron microscope (Lehmann et al., 2024).

The synergistic or additive interaction between the purified fengycin and bacillomycin D was determined by the Loewe additivity model (Luna-Bulbarela et al., 2018). An isobologram was constructed based on the concentrations of the two lipopeptides, and the combination index (CI) was calculated using the following equation:

$$\text{CI}\;=\;\frac{CA}{\text{ICA}}+\frac{CB}{\text{ICB}}$$

where: *C*_A_ and *C*_B_ are the concentrations of the fengycin and bacillomycin D in the combination required to achieve a specific level of inhibition (*e.g.*, 70, 80, 90 %), and IC_A_ and IC_B_ are the concentrations of each lipopeptide alone required to achieve the same effect. A CI value < 1 indicates synergism, CI = 1 indicates an additive effect, and CI > 1 indicates antagonism.

### 2.5 Analysis and bioactivity of volatile organic compounds (VOCs)

The bioactivity assay of *B. velezensis* GFB08 VOCs was tested using the double-sealed plate method (90 mm Petri dish), following recent publications (Xing et al., 2023). Briefly, 100 μL of GFB08 overnight culture (10^7^ CFU/mL) was spread on nutrient agar (NA) medium. After two days of incubation, hyphal disks (4 mm in diameter) of *Colletotrichum spaethianum* CO15–2, *C. circinans* 30–1, and *Stemphylium vesicarium* SV02 were placed in the center of the PDA medium. Two plates were sealed together with parafilm and incubated at 25 °C for 7 days. NA plates spread with 100 μL LB medium were used as controls. The experiments were performed using five biological replicates, and the inhibition rate was calculated as described in Section 2.4.

GC–MS analysis of VOCs performed as described in Chen et al. (2023) and Zhao et al. (2024). For sample preparation, 100 μL of *B. velezensis* GFB08 overnight culture was spread on NA medium and incubated for two days. NA plates spread with 100 μL of LB medium were used as controls. Three independent biological replicates were prepared for VOC analysis, and two technical replicates were performed for each biological replicate. The equipment was equipped with a HP-5MS column (30 *m* × 0.25 mm × 0.25 µm). The GC temperature program was initiated at 40 °C for 3.5 min, increased at 10 °C/min to 280 °C, and held for 2.5 min. The column flow rate was maintained at 1 mL/min. Mass spectra were acquired using electron impact (EI) ionization at 70 eV. Retention indices (RI) were calculated for the HP5-MS column using an n-alkane (C7–C40) standard series.

The volatile compound standards were purchased from Supelco (Sigma-Aldrich, USA), and GC–MS confirmed the purity of each compound. The bioactivity of individual VOC standards was evaluated using the fumigation method as detailed in Xing et al. (2023). Afterward, 90 mm Petri dishes were filled with 12 mL of PDA, and hyphal disks (4 mm diameter) of *Colletotrichum spaethianum* CO15–2 were placed in the center. Individual compounds were pipetted onto sterile filter paper discs (13 mm diameter) to obtain the following headspace concentrations: 0.1, 0.2, 0.4, 0.8 μL/mL. The PDA plates were then sealed with parafilm and incubated at 25 °C for 7 days. Control plates consisted of filter paper discs treated with an equivalent volume of ddH₂O. The experiments were performed in triplicate, and the inhibition rate was calculated as described in Section 2.4.

### 2.6 Plant growth promotion (PGP) assays

The PGP effect of *B. velezensis* GFB08 was evaluated at two stages: a seed soaking assay (seed stage) and a seedling treatment assay (seedling stage).

For inoculation, the GFB08 suspension was prepared by centrifuging an overnight culture at 4000 × *g* for 10 min. The resulting cell pellet was then resuspended in sterile water to achieve a concentration of 10^7^ CFU/mL (Li et al., 2024).

For seed soaking assay, Welsh onion seeds (SV-560) were purchased from Known-You Seed Co., Taiwan. In total, 50 seeds were surface-sterilized using 75 % ethanol for 2 min, followed by 2 % sodium hypochlorite for 5 min, then rinsed three times with sterile water. After sterilization, the seeds were soaked in either *B. velezensis* GFB08 suspension or sterile water as a control (CK) for 24 h. The treated seeds were then transferred to sterile, moist tissue paper to germinate. After three days, the germinated seeds were transplanted into pots (4.25 × 4.25 × 7 cm) containing soilless medium (Klasmann-Deilmann, Germany) and grown in a greenhouse maintained at 20–25 °C with a 13-h/11-h light/dark photoperiod. Plants were treated weekly with 30 mL of *B. velezensis* GFB08 suspension or sterile water as a control. Shoot height, root length, fresh weight, and dry weight were measured after one month of cultivation. Each treatment included five replicates and was repeated in two independent experimental rounds.

For the seedling assay, Welsh onion mature seedlings were obtained from Welsh onion fields in Sanxing, Taiwan. The seedlings were pruned to 10 cm in height, then transplanted into pots containing the same soilless medium, under identical greenhouse conditions as described earlier in Section 2.6. Plants were treated weekly with 30 mL of *B. velezensis* GFB08 suspension or sterile water. At the four-leaf stage (after 4–5 weeks of growth), shoot height, root length, fresh weight, and dry weight were recorded. Each treatment included five replicates and was repeated in two independent experimental rounds.

### 2.7 Statistical analysis

All data were analyzed using *t*-tests or one-way ANOVA followed by Tukey's HSD test, with R software version 4.3.0. A P-value < 0.05 was considered statistically significant. Data visualizations were generated using GraphPad Prism (v. 9.5.1).

## 3 Results

### 3.1 Genome features of Bacillus velenzsis GFB08

**Fig. 1:**
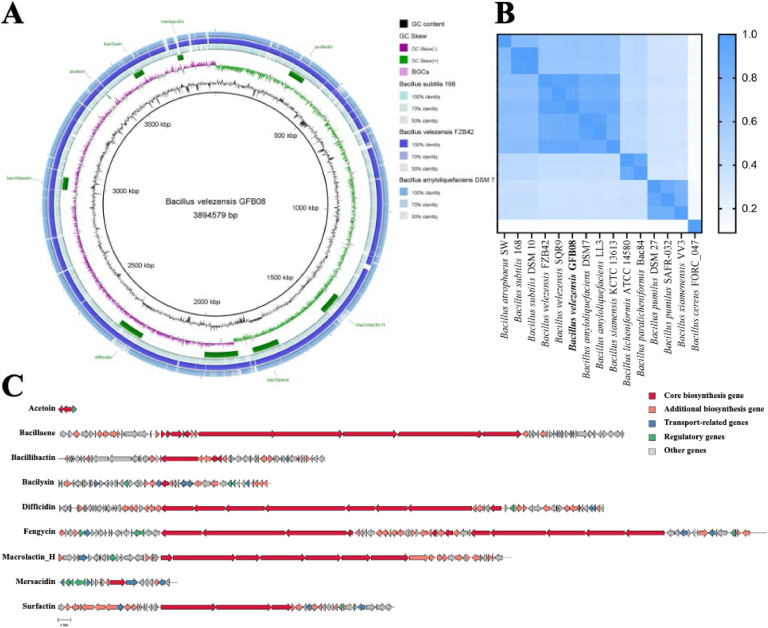
Genome features of *Bacillus velezensis* GFB08. (A) Circular genome map constructed with BRIG v0.95. From the innermost to the outermost layers: GC content, GC skew, secondary metabolite biosynthetic gene clusters, and whole-genome BLAST comparisons with *B. subtilis* 168, *B. velezensis* FZB42, and *B. amyloliquefaciens* DSM7. (B) The heatmap based on the ANIb value between *B. velezensis* GFB08 and related *Bacillus* species. The color bar represents ANIb value between any two strains, starting from white to blue (0 or 100 %) (C) Biosynthetic gene clusters (BGCs) predicted in *B. velezensis* GFB08.

To characterize the genome of *Bacillus velezensis* GFB08, a hybrid sequencing approach was executed, combining Nanopore long-reads (153,361 reads; 182x coverage) and Illumina short-reads (8895,668 reads; 359x coverage). The resulting assembly comprises a single circular chromosome of 3894,579 bp with a GC content of 46.64 %, as shown in Fig. 1A and Supplementary Table S2. BUSCO assessment against the Bacillales dataset received a high level of genome assembly completeness, with 99.8 % of single-copy genes (Supplementary Table S3). Genome annotation using the NCBI PGAP pipeline predicted a total of 3853 genes, including 3735 protein-coding sequences (CDSs), 27 rRNA genes, and 86 tRNA genes (Supplementary Table S2).

The taxonomic identity of strain GFB08 was confirmed via ANI analysis against related *Bacillus* species, as recently published (Dunlap et al., 2016). GFB08 exhibited high ANIb values (>98 %) with other *B. velezensis* strains, including the ex-type strain of *B. velezensis* FZB42, verifying its classification within this species (Fig. 1B).

The genome of *B. velezensis* GFB08 reveals notable antimicrobial potential. AntiSMASH v7.0.0 anticipated eight BGCs, including well-known antifungal lipopeptides such as surfactin, fengycin, and bacillomycin, together with antibacterial compounds like bacilysin, difficidin, and macrolactin (Fig. 1C). Furthermore, the PGAP annotation discovered the presence of the acetoin gene cluster (*budA, alsS, alsR*), which is involved in the synthesis of a volatile organic compound (VOC) with reported antifungal and plant growth-promoting activity (Fincheira et al., 2016).

### 3.2 Comparative genomics

**Fig. 2:**
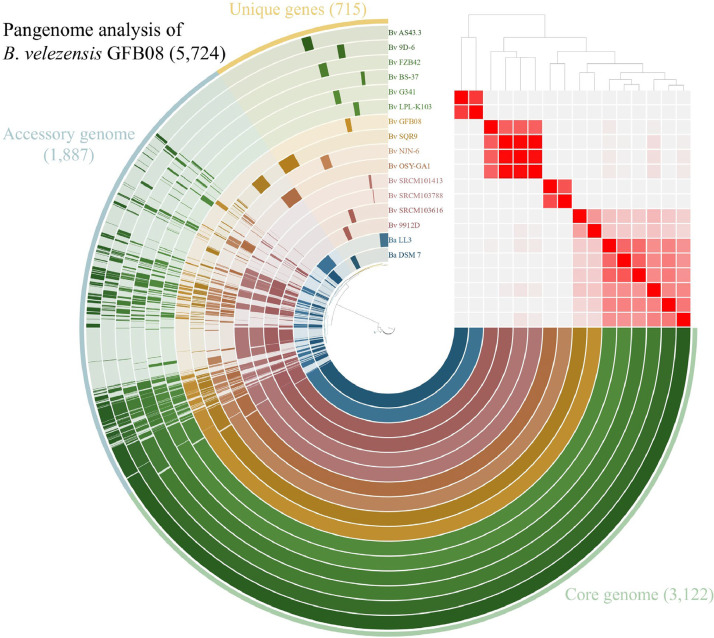
Pan-genome analysis of *Bacillus velezensis* GFB08 and closely related species. The layers represent individual genomes organized based on ANIb value (97–100 %). Within the layers, dark and light colors indicate the presence or absence of gene clusters, respectively. In the outermost layer, core genome, accessory genome, and unique genes are highlighted in green, blue, and yellow, respectively.

To further elucidate the genomic content and variability of *Bacillus velezensis* GFB08 compared to other *Bacillus velezensis* genomes, a pangenome analysis was conducted. This analysis included the genome of GFB08 along with thirteen other *B. velezensis* strains and two *B. amyloliquefaciens* strains, and the strain details are provided in Supplementary Table S1. The pangenome of these 16 strains comprised a total of 5724 gene clusters (GCs) (Fig. 2). These GCs were categorized into three groups based on their occurrences across the genomes. The core genome (100 % occurrence) consisted of 3122 GCs. The large core genome highlights the genomic similarity between GFB08 and other *B. velezensis* strains, especially SQR9 (accession no NZ_CP006890.1). The accessory genome included 1887 GCs found in some but not all genomes, representing a key source of genomic variation. Finally, 715 gene clusters were identified as unique to a single genome and of these, *B. velezensis* GFB08 possessed 30 singletons. Functional annotation showed putative functions for eight of them, including defense mechanisms, DNA modification, transcriptional regulation, and specific metabolic processes (Supplementary Table S4). Notably, further analysis using BLASTp revealed that three of these unique gene clusters in GFB08 (GC_00005460, GC_00005385, and GC_00005678) showed homology to *mrsA, mrsM*, and *mrsT*, respectively. These genes are known to be involved in the biosynthetic gene cluster (BGC) for mersacidin, a type II lantibiotic.

The genetic variations within secondary metabolite biosynthetic gene clusters (BGCs) were further assessed. A known challenge in analyzing *Bacillus* BGCs is that adjacent clusters, such as the bacillomycin and fengycin clusters, are often predicted as a single merged region (Koumoutsi et al., 2004). To address this, a modified version of antiSMASH, as described by Steinke et al. (2021), was employed to accurately separate neighboring clusters. The fengycin family is known for its structural diversity, and among the 16 analyzed strains, fengycin BGCs could be grouped into two categories. In total, eight strains including GFB08, contained complete sets of core biosynthesis genes (*fenA* to *fenE*), while others possessed incomplete BGCs, missing up to three biosynthesis genes (Fig. S1).

Comparative analysis revealed that the examined *B. velezensis* strains possess BGCs encoding for iturin, bacillomycin D, and bacillomycin L (Fig. S2). Specifically, *B. velezensis* GFB08 harbors a complete bacillomycin D BGC (*bmyA*-*bmyD*). Synteny analysis further revealed that these fengycin and bacillomycin D clusters are highly conserved with those of *B. velezensis* SQR9 and FZB42. These findings provide the genomic basis for the strain's capacity to produce antifungal lipopeptides, particularly fengycin and bacillomycin D.

### 3.3 Isolation, identification, and bioactivity assay of lipopeptides

The antifungal potential of *Bacillus velezensis* GFB08 was initially evaluated using its cell-free supernatant against three Welsh onion foliar pathogens: *Colletotrichum spaethianum* CO15–2, *C. circinans* CO30–1, and *Stemphylium vesicarium* SV02. The cell-free supernatant of GFB08 exhibited inhibitory activity, with IC_50_ values of >2000 mg/L for *C. spaethianum*, 1802.99 mg/L for *C. circinans*, and 258.63 mg/L for *S. vesicarium* (Table 1 and Fig. S3–S5). To further identify active antifungal components, several extraction methods were employed.

**Table 1:** Bioactivity assay of cell-free supernatant, crude extracts, and purified lipopeptides against Welsh onion foliar pathogens.

	IC_50_ (mg/L)		
	*C. spaethianum*	*C. circinans*	*S. vesicarium*
Cell-free supernatant	>2000	1802.99	258.63
HP20	>2000	777.9	493.12
Ethyl acetate	1173.46	853.57	466.28
Ammonium sulfate precipitation	469.31	144.03	<125
Acid precipitation	259.57	134.2	<125
Fengycin	61.03	50.97	26.09
Bacillomycin D	58.74	>100	>100

Comparative bioassays of these crude extracts revealed significant differences in their overall antifungal activity (Table 1 and Fig. S3–S5). Acid precipitation resulted in extracts with the lowest IC_50_ values against the three tested pathogens: 259.57 mg/L for *C. spaethianum*, 134.2 mg/L for *C. circinans*, and <125 mg/L for *S. vesicarium*. Extracts from ammonium sulfate precipitation also showed potent inhibitory activity, with IC_50_ values of 469.31 mg/L, 144.03 mg/L, and <125 mg/L for *C. spaethianum, C. circinans*, and *S. vesicarium*, respectively. In contrast, extracts obtained using HP20 resin adsorption (IC_50_ values ranging from 493.12 mg/L to >2000 mg/L) and ethyl acetate extraction (IC_50_ values ranging from 466.28 mg/L to 1173.46 mg/L) exhibited considerably lower antifungal efficacy.

Given the variable bioactivities of the four extraction methods, LC-MS analysis was performed on each extract to identify potential bioactive compounds. Principal component analysis (PCA) of the untargeted LC-MS data showed the metabolite profile of the EA extract differed significantly from those of the other three extracts, as shown in Supplementary Fig. S6A. Following the genome-based prediction of the corresponding biosynthetic gene clusters in GFB08, the analysis then focused on a targeted search for surfactin and fengycin using their extracted ion chromatograms (EICs). Surfactin was found to be most abundant in the EA extract, which exhibited the lowest bioactivity, and was present in lower relative levels in the other three extracts (Supplementary Fig. S6B). This distribution suggested that surfactin is unlikely to be the primary compound responsible for the antifungal activity. In contrast, fengycin was predominantly detected in the highly active acid precipitation and ammonium sulfate precipitation extracts, but was present at much lower levels or absent in the EA and HP20 extracts (Fig. S6B). This pattern supports the role of fengycin as a potential contributor to the potent antifungal bioactivity observed in extracts from acid precipitation and ammonium sulfate precipitation.

**Fig. 3:**
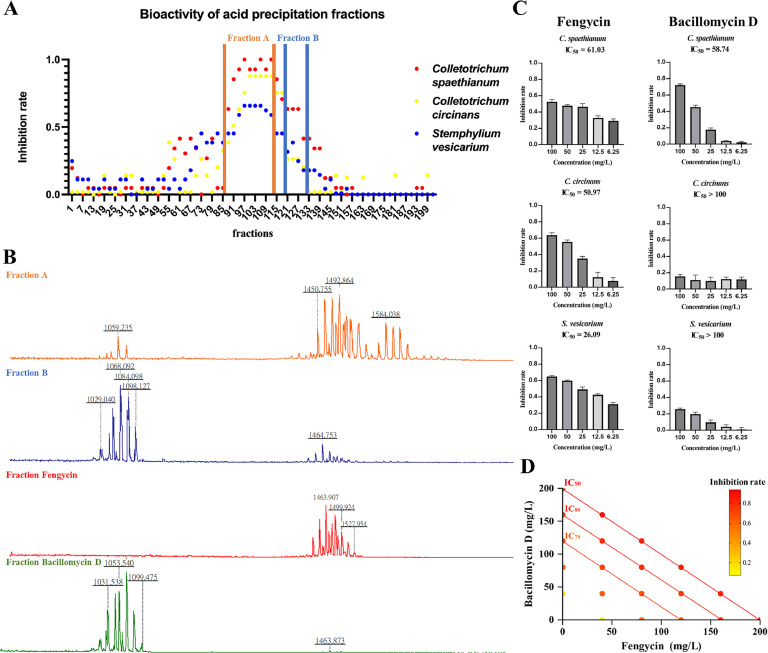
Isolation and Identification of Bioactive Lipopeptides. (A) Bioactivity screening of every third fraction against Welsh onion foliar pathogens. Fraction A (88–115) showed the most potent bioactivity across all three pathogens, while fraction B (116–130) exhibited partial bioactivity. (B) MALDI-TOF-MS detection of metabolites in fraction A, B, fengycin, and bacillomycin D. (C) Bar chart quantifying the percentage of mycelial growth inhibition of purified fengycin and bacillomycin. (D) Loewe additive test for purified fengycin and bacillomycin D against *C. spaethianum*.

Subsequently, the crude extract obtained from acid precipitation was fractionated using Sephadex LH-20 column chromatography. A total of 201 fractions were collected and evaluated for both antifungal bioactivities and metabolite profiling. Initial screening revealed that fractions 88–115 exhibited the most potent antifungal activity against all three tested pathogens (Fig. 3A and Fig. S7). MALDI-TOF-MS analysis of these active fractions showed dominant signals of fengycin (Fig. 3B). Fractions 116–130 displayed moderate bioactivity and were characterized by strong bacillomycin D signals, with only trace amounts of fengycin detected.

To further purify these lipopeptides, a second round of Sephadex LH-20 column chromatography was performed on the active fractions. Subsequent MALDI-TOF-MS and LC-MS/MS analyses confirmed the identities and composition of these purified fractions (Supplementary Table S5; Fig. S8). The fengycin fraction consisted of a mixture of C15, C16, and C17-fengycin A, along with C15, C16, and C17-fengycin B, which differ by alanine (Ala) and valine (Val) residues at position 6 of the peptide ring. The bacillomycin D fraction was determined to contain C14 and C15-bacillomycin D isoforms.

Bioassays of the purified lipopeptides revealed that fengycin exhibited vigorous antifungal activity against all three pathogens, with IC_50_ values of 61.03 mg/L for *C. spaethianum*, 50.97 mg/L for *C. circinans*, and 26.09 mg/L for *S. vesicarium* (Fig. 3C and Fig. S9). These results strongly indicate fengycin as the primary bioactive compound responsible for the observed antifungal activity in the present study. Interestingly, the purified bacillomycin D effectively inhibited growth only against *C. spaethianum* (IC_50_ = 58.74 mg/L), and showed no significant bioactivity against *C. circinans* and *S. vesicarium* (IC_50_ > 100 mg/L).

The interaction between purified fengycin and bacillomycin was assessed against *C. spaethianum* based on the Loewe additivity model. For all tested combinations of fengycin and bacillomycin D that resulted in 70 %, 80 %, and 90 % inhibition, the calculated CI values were equal to 1, indicating an additive effect of these two lipopeptides. Isobologram analysis further confirmed this finding, as all effective combinations lay directly on the lines of additivity for IC_70_, IC_80_, and IC_90_ (Fig. 3D). These results suggest that fengycin and bacillomycin D act independently against *C. spaethianum*, while exhibiting comparable levels of antifungal activity.

Scanning electron microscopy (SEM) was used to visualize the morphological changes induced by compounds with significant antifungal activity. In the untreated controls, hyphae of all three pathogens appeared slender with intact cell structures (Fig. S10A–C). In contrast, treatment with fengycin caused severe morphological alterations. The hyphae of *C. spaethianum, C. circinans*, and *S. vesicarium* exhibited irregular expansion and rupture, ultimately leading to cell death (Fig. S10D-F). When treated with bacillomycin D, *C. spaethianum* hyphae also showed clear signs of damage, including expansion (Fig. S10G). However, the degree of expansion was comparatively lower than that caused by fengycin, and hyphal ruptures were rarely observed. These SEM observations indicate that fengycin induces extensive and severe physical damage to the hyphae of all three tested pathogens, consistent with its potent broad-spectrum antifungal activity. Bacillomycin D also causes visible hyphal damage to *C. spaethianum*, aligning with its species-specific activity against this pathogen. However, its effects appear less severe than those of fengycin at the tested concentrations.

### 3.4 Collection, identification, and bioactivity assay of VOCs

**Fig. 4:**
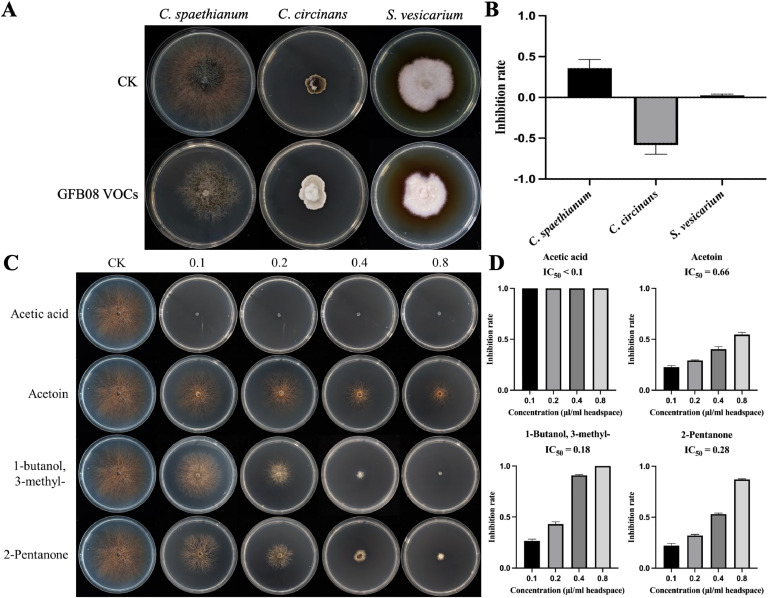
Collection, identification, and bioactivity assay of VOCs. (A) Representative photograph showing the effect of GFB08 VOCs on the mycelial growth of *C. spaethianum, C. circinans*, and *S. vesicarium.* (B) Bar chart quantifying the percentage of mycelial growth inhibition. (C) Representative photograph showing the effect of VOC standards on the mycelial growth of *C. spaethianum, C. circinans*, and *S. vesicarium.* (D) Bar chart quantifying the percentage of mycelial growth inhibition.

The antifungal activity of VOCs produced by *B. velezensis* GFB08 was assessed using a double sealed-plate method. VOCs released by *B. velezensis* GFB08 inhibited the growth of *C. spaethianum* but did not affect the growth of *S. vesicarium*. Interestingly, VOCs appeared to stimulate the growth of *C. circinans* under the tested conditions (Fig. 4A and B).

To identify the specific VOCs responsible for these observations, GC–MS analysis was performed on the VOCs released by *B. velezensis* GFB08 across three biological replicates. This analysis identified acetone, acetic acid, 2-pentanone, acetoin, 3-methyl-1-butanol, 2-acetylthiazole, and 2-methoxyphenol as the major VOCs released by *B. velezensis* (Table 2 and Fig. S11). Based on previous reports of antifungal activity, standards of acetic acid, 2-pentanone, acetoin, and 3-methyl-1-butanol were selected for further validation (Asghar and Kataoka, 2025; Song et al., 2022; Yuan et al., 2025).

**Table 2:** GC–MS identification of VOCs released by *Bacillus velezensis* GFB08.

Compound	RT	KI	RKI	Identification	References of antifungal activity
Acetone	5.820	NA	503	MS	
Acetic acid	6.578	NA	NA	MS, ST	Yuan et al., 2025
2-Pentanone	8.226	688	686	MS, ST	Asghar and Kataoka, 2025
Acetoin	8.649	709	720	MS, ST	Song et al., 2022
3-Methyl-1-butanol	9.112	731	730	MS, ST	Song et al., 2022
2-Acetylthiazole	14.887	1028	1021	MS	
2-Methoxyphenol	16.052	1097	1096	MS	

Notes: RT, retention time; KI, Kovats retention index in this study; RKI, Kovats retention index in references; MS, GC–MS; ST, compound standards; NA means the KI value for the compound cannot be calculated or is not reported in the references.

GC–MS confirmed the purity of each selected standard, and their individual antifungal activities were tested by applying them to sterile filter paper discs, with headspace concentrations ranging from 0.1 to 0.8 µL/mL (Fig. 4C and D). All four VOC standards inhibited the growth of *C. spaethianum*. Notably, acetic acid completely inhibited mycelial growth at 0.1 µL/mL. 3-Methyl-1-butanol also achieved 100 % inhibition at 0.8 µL/mL, with an IC₅₀ of 0.18 µL/mL. Both 2-pentanone and acetoin exhibited partial inhibition at 0.8 µL/mL, with IC₅₀ values of 0.28 µL/mL and 0.66 µL/mL, respectively. These findings suggest that, in addition to producing antifungal lipopeptides, *B. velezensis* GFB08 also releases VOCs that contribute to its overall biocontrol potential, particularly against *C. spaethianum*.

### 3.5 Plant growth promotion assay

The PGP effect of *B. velezensis* GFB08 was evaluated at two stages: the seed stage and the seedling stage. For the seed stage, a seed soaking assay was performed, in which surface-sterilized Welsh onion seeds were soaked in the bacterial suspension. For the seedling stage, the bacterial suspension was directly applied to the seedlings, as described earlier in the Materials and Methods section. Significant plant growth promotion ability was observed in the seed soaking assay. This seed treatment resulted in significantly increased shoot height, fresh weight, and dry weight after one month of cultivation when compared to the untreated control (Fig. S12).

However, *B. velezensis* GFB08 applied to the seedling stage did not show significant differences in growth parameters (shoot height, root length, fresh weight, and dry weight) compared to the control group at the four-leaf stage (Fig. S13). Collectively, these results suggest that *B. velezensis* exhibits significant plant growth-promoting activity primarily at the seed stage.

## 4 Discussions

Unlike most previous work that examined either the genomic or metabolite dimension alone, our study integrates targeted chemistry, genomic context, and functional bioassays for a single biocontrol strain of *Bacillus velezensis*. This multidimensional approach provides a mechanistic and more complete understanding of biocontrol potential.

As integrated pest management (IPM) strategies gain importance in sustainable agriculture, microbial biocontrol agents such as *B. velezensis* have received increasing attention for their ability to suppress plant pathogens through diverse mechanisms (Kenfaoui et al., 2024). However, most studies have characterized general antifungal properties or examined lipopeptides and VOCs independently. Our work addresses the foliar disease complex of Welsh onion, a challenging combination of *Stemphylium vesicarium, Colletotrichum spaethianum*, and *C. circinans* that requires broad-spectrum efficacy. By systematically elucidating how lipopeptides and VOCs function synergistically, we provide the first mechanistic characterization of a biocontrol agent against the emerging threat of *S. vesicarium* in Taiwan, addressing a critical gap for sustainable agriculture in the region.

In our previous work, *B. velezensis* GFB08 was isolated from healthy Welsh onion leaves and characterized for its potential to control foliar pathogens (Wang et al., 2023). In the present study, we further elucidated its antifungal mechanisms, demonstrating that GFB08 produces lipopeptides and volatile organic compounds (VOCs) with potent inhibitory activity against multiple pathogens, in addition to exhibiting plant growth–promotion effects.

To explore the genetic basis of its biocontrol effects, we conducted a detailed genome analysis of *B. velezensis* GFB08. Phylogenetic analysis, supported by average nucleotide identity analysis, firmly classified GFB08 as a member of the *B. velezensis*. Recent studies highlight the significant genetic diversity within *B. velezensis* strains, including substantial variation in biosynthetic gene clusters (BGCs) and the detection of multiple putative novel BGCs (Bach et al., 2025; Dhanalakshmi and Rajendhran, 2024). Pangenome analyses revealed an open pangenome for *B. velezensis*, indicating this species exhibits dynamic gene content, where individual strains often possess unique genes not found in others (Sousa et al., 2025). Our comparative genomic analysis of strain GFB08 align with this understanding. Despite this overall similarity, several unique gene clusters were detected, including homologous of key genes (*mrsA, mrsM, mrsT*) in the mersacidin biosynthetic pathway. Mersacidin is a potent antibacterial peptide that inhibits cell wall biosynthesis (Schmitz et al., 2006). This biosynthetic pathway is also located within a genomic island in *B. amyloliquefaciens* strain Y2, which may explain its variable presence among *Bacillus* strains (He et al., 2012).

*B. velezensis* strains have received growing interest for their capacity to produce bioactive secondary metabolites (Kenfaoui et al., 2024). *B. velezensis* FZB42, which was isolated from sugar beet root, is a well-studied biocontrol strain that devotes approximately 8.5 % of its genome to the synthesis of antibiotics and siderophores, emphasizing the importance of secondary metabolites in this species (Chen et al., 2007). The diversity of BGCs in *B. velezensis* contributes to the production of a broad array of secondary metabolite variants. The iturin lipopeptide family, for instance, exhibits structural diversity, including variants such as iturin, bacillomycin D, bacillomycin F, bacillomycin L, and mycosubtilin, which differ in the amino acid composition of their cyclic heptapeptide structure (Dunlap et al., 2019). This diversity reflects underlying variations in their corresponding BGCs. In particular, the BGCs responsible for the production of fengycin (*fenABCDE*) and bacillomycin D (*bmyDABC*) were found to be highly conserved across these strains, underscoring a shared genetic foundation for their biocontrol capabilities.

The investigation into the lipopeptides produced by *B. velezensis* GFB08 revealed distinct antifungal activities and interactions for fengycin and bacillomycin D. Fengycin is known to degrade cell wall integrity and cell membranes, ultimately leading to leakage (Chen et al., 2025). Bacillomycin D functions by affecting the surface tension of the fungal cell membrane and forming micropores (Wang et al., 2024). The potent antifungal activity is supported by our SEM observation, which confirmed that both lipopeptides induce irregular expansion and rupture to fungal hyphae (Fig. S10) . The results demonstrated that GFB08-produced fengycin exhibits broad-spectrum efficacy, effectively inhibiting all three Welsh onion foliar pathogens used in this study. In contrast, bacillomycin D from GFB08 displayed a species-specific effect, showing potent inhibition against *C. spaethianum* but limited efficacy against *C. circinans* and *S. vesicarium*. This pathogen-dependent efficacy is a known feature of lipopeptides produced by *B. velezensis*. For instance, bacillomycin D has been identified as a major antifungal lipopeptide produced by *B. velezensis* SQR9 against *Fusarium oxysporum*. At the same time, fengycin also contributed to antifungal activities against *Verticillium dahliae, F. oxysporum, F. solani*, and *Phytophthora parasitica* (Li et al., 2014). Furthermore, the interaction between fengycin and bacillomycin D produced by GFB08 against *C. spaethianum* was determined to be additive in this study. This observation contrasts with some previous reports. For example, in *B. velezensis* FZB42, fengycin and bacillomycin D have been described to act synergistically against *F. oxysporum* (Koumoutsi et al., 2004). Similarly, Luna-Bulbarela et al. (2018) reported that bacillomycin D homologues produced by *B. velezensis* 83 exhibited a synergistic effect on the antifungal activity against *Colletotrichum gloeosporioides*. Our finding of an additive interaction between fengycin and bacillomycin D reveals a key mechanistic difference from commercial strains like FZB42 and SQR9. This crucial lipopeptide interaction is highly specific and dependent on both the *Bacillus* strain and the susceptibility of the target pathogen, which underscores the importance of characterizing these mechanisms on a case-by-case basis.

In addition to lipopeptides, VOCs produced by *B. velezensis* GFB08 also exhibited species-specific antifungal activity. In double-sealed plate assays, these VOCs significantly inhibited the growth of *C. spaethianum* but showed no inhibitory effect against *S. vesicarium*. Remarkably, VOCs released by *B. velezensis* even promoted the growth of *C. circinans*. Further GC–MS analysis and bioassays identified acetic acid, 2-pentanone, acetoin, and 3-methyl-1-butanol as major antifungal VOCs produced by *B. velezensis* GFB08. Acetic acid is a widely recognized antifungal compound that effectively inhibits a broad spectrum of fungal pathogens, including *Botrytis cinerea, Botryosphaeria dothidea*, and *Colletotrichum gloeosporioides* (Kang et al., 2003; Toral et al., 2021; Yuan et al., 2025). Due to its higher pKa value, a significant portion of acetic acid exists in undissociated form, allowing for greater membrane permeability resulting in strong antifungal activity (Liu et al., 2022). Many studies have also reported 2-pentanone, acetoin, and 3-methyl-1-butanol as common antifungal VOCs produced by *B. velezensis* (Calvo et al., 2020; Lim et al., 2017; Ling et al., 2022). While many studies highlight the inhibitory effects of bacterial VOCs on fungal growth, exceptions where VOCs from certain bacteria stimulate fungal growth are also reported. Briard et al. (2016) found that sulfur-containing VOCs such as dimethyl sulfide and dimethyl disulfide produced from *Pseudomonas aeruginosa* enhance the growth of *Aspergillus fumigatus* under sulfur-limiting conditions. Similarly, bacterial VOCs such as 2,5-diisopropylpyrazine significantly stimulated the mycelial growth of *Pleurotus ostreatus* and *P. eryngii*, but not other mushroom species, indicating that VOC responses are often species-specific (Orban et al., 2023). Thus, similar to lipopeptides, the VOCs produced by *B. velezensis* GFB08 exhibit species-specific effects. The unexpected growth promotion of *C. circinans* highlights the complexity of bacterial–fungal interactions, potentially involving hormetic responses or enhanced nutrient uptake. The underlying mechanisms require further investigation.

In the plant growth promotion assay, *B. velezensis* GFB08 exhibited significant growth-promoting activity, particularly at the seedling stage. This observation is consistent with previous reports showing that the plant growth-promoting (PGP) effects of rhizobacteria are often more significant during the early stages of plant development, such as seed germination and seedling establishment (Gholami et al., 2009; Pérez-García et al., 2023). For example, *B. velezensis* BAC03 showed the highest enhancement of radish growth when applied 10 days after planting, while having a minor effect when applied 30 days after planting (Meng et al., 2016). The non-significant result in the seedling assay may be due to the plant's physiological state. At the four-leaf stage, Welsh onions begin to tiller, and this physiological shift could mask the PGP effect observed at germination. Nevertheless, our results identified potential PGP activity, as the GFB08 genome harbors the acetoin biosynthesis gene cluster and GC–MS analysis confirmed acetoin production which is a VOC well-known for its plant growth-promoting capabilities (Calvo et al., 2020). While additional time-point analysis may further clarify the PGP mechanism, the current findings already align with early-stage PGP activity patterns reported for other *Bacillus* strains, suggesting this trait is most relevant at germination and seedling establishment.

## 5 Conclusion

In summary, this study provides the first integrative genomic–metabolite–functional characterization of *B. velezensis* GFB08, revealing a unique mersacidin-associated BGC, additive lipopeptide interactions, and pathogen-specific VOC effects. Together, these findings underscore the importance of mechanistic, strain-level studies for designing effective biocontrol strategies against complex foliar disease systems such as those affecting Welsh onion in Taiwan. This work not only lays the foundation for developing GFB08 as a field-applicable component of integrated pest management but also demonstrates a framework for characterizing biocontrol agents with multi-target efficacy.

## CRediT authorship contribution statement

**Yi-Tun Cho**: Methodology, Investigation, Writing – original draft. **Hieng-Ming Ting**: Writing – review & editing, Supervision. **Bo-Wei Wang**: Methodology, Investigation, Data curation. **Yi-Chen Tsai**: Methodology, Resources. **Hao-Yung Wang**: Methodology, Data curation. **Yu-Liang Yang**: Writing – review & editing, Supervision. **Hiran A. Ariyawansa**: Writing – review & editing, Supervision.

## Funding

This research was supported by the Ministry of Agriculture (Grant number: 111AS −1.3.2-AS-aN, 112AS-1.3.2-AS-aF, 113AS-1.3.2-AS-28) and National Science and Technology Council (Grant number: 112–2313-B-002–027-MY3, 113–2321-B-002–043-, 113-2628B-002-019-MY3, 114-2321-B-002-020 -).

## Declaration of competing interest

The authors declare that they have no known competing financial interests or personal relationships that could have appeared to influence the work reported in this paper.

## Data Availability

Data will be made available on request.

## Appendix Supplementary materials

Image, application 1
