# Supplementary material for: Integrating genomics and targeted metabolite profiling to elucidate disease-suppression mechanisms of Bacillus velezensis GFB08
Source: Curr Res Microb Sci. 2025 Oct 30;9:100503. doi: 10.1016/j.crmicr.2025.100503 (PMC12637390; doi:10.1016/j.crmicr.2025.100503)
Supplement: Supplementary file 1 [file mmc1.docx]

**Table S1.** Details of *Bacillus* genomes used in this study.

| **Species** | **Strain** | **Accession** |
| --- | --- | --- |
| ***B. atrophaeus*** | *Bacillus atrophaeus* SW | NZ_CP154443.1 |
| ***B. subtilis*** | *Bacillus subtilis* subsp. *subtilis* str. 168 | NC_000964.3 |
|  | *Bacillus subtilis* DSM 10 | NZ_CP034484.1 |
| ***B. velezensis*** | *Bacillus velezensis* FZB42 | NC_009725.1 |
|  | *Bacillus velezensis* SQR9 | NZ_CP006890.1 |
|  | *Bacillus velezensis* AS43.3 | NC_019842.1 |
|  | *Bacillus velezensis* 9D-6 | NZ_CP020805.1 |
|  | *Bacillus velezensis* SRCM103788 | NZ_CP035399.1 |
|  | *Bacillus velezensis* SRCM103616 | NZ_CP035410.1 |
|  | *Bacillus velezensis* SRCM101413 | NZ_CP021890.1 |
|  | *Bacillus velezensis* OSY-GA1 | NZ_CP031880.1 |
|  | *Bacillus velezensis* G341 | NZ_CP011686.1 |
|  | *Bacillus velezensis* BS-37 | NZ_CP023414.1 |
|  | *Bacillus velezensis* LPL-K103 | NZ_CP039380.1 |
|  | *Bacillus velezensis* NJN-6 | NZ_CP007165.1 |
|  | *Bacillus velezensis* 9912D | NZ_CP017775.1 |
| ***B. amyloliquefaciens*** | *Bacillus amyloliquefaciens* DSM 7 | NC_014551.1 |
|  | *Bacillus amyloliquefaciens* LL3 | NC_017190.1 |
| ***B. siamensis*** | *Bacillus siamensis* KCTC 13613 | NZ_AJVF00000000.1 |
| ***B. licheniformis*** | *Bacillus licheniformis* ATCC 14580 | NZ_CP140161.1 |
| ***B. paralicheniformis*** | *Bacillus paralicheniformis* Bac84 | NZ_CP023665.1 |
| ***B. pumilus*** | *Bacillus pumilus* DSM 27 | NZ_CP046130.1 |
|  | *Bacillus pumilus* SAFR-032 | NC_009848.4 |
| ***B. xiamenensis*** | *Bacillus xiamenensis* VV3 | NZ_CP017786.1 |
| ***B. cereus*** | *Bacillus cereus* FORC_047 | NZ_CP017060.1 |

**Table S2.** Genome features of *Bacillus velezensis* GFB08.

| GFB08 genome feature | |
| --- | --- |
| Contigs | 1 |
| Total length | 3,894,579 |
| GC (%) | 46.64 |
| Total genes | 3,853 |
| CDS | 3,735 |
| Genes (RNA) | 118 |
| rRNAs | 27 |
| tRNAs | 86 |

**Table S3.** BUSCO genome assembly completeness assessment of *Bacillus velezensis* GFB08.

| GFB08 BUSCO completeness | | | | |
| --- | --- | --- | --- | --- |
|  | S | D | F | M |
| Bacteria | 100 | 0 | 0 | 0 |
| Firmicutes | 100 | 0 | 0 | 0 |
| Bacilli | 99.7 | 0.3 | 0 | 0 |
| Bacillales | 99.8 | 0 | 0.2 | 0 |

Notes：S，Single-copy；D，Duplicated；F，Fragmented；M，Missing。

**Table S4.** COG Category of *Bacillus velezensis* GFB08 unique gene clusters

| Gene cluster | COG Category | | COG Category Description | Putative Function |
| --- | --- | --- | --- | --- |
| GC_00005460 | H | | Coenzyme transport and metabolism | Phosphopantothenoylcysteine synthetase/decarboxylase CoaBC (CoaBC) (PDB:3QJG) |
| GC_00005593 | L | Replication, recombination, and repair | | DNA-cytosine methylase (Dcm) (PDB:3LX6) |
| GC_00005303 | Q | Secondary metabolites biosynthesis, transport, and catabolism | | Dienelactone hydrolase (DLH) (PDB:4ZV9) |
| GC_00005489 | R | General function prediction only | | Penicillin-binding protein-related factor A, putative recombinase (YotM) (PDB:1RZN) |
| GC_00005093 | T\|K | Signal transduction mechanisms\|Transcription | | DNA-binding response regulator, OmpR family, contains REC and winged-helix (wHTH) domain (OmpR) (PDB:1XHF) |
| GC_00005183 | V | Defense mechanisms | | ABC-type bacteriocin/lantibiotic exporters, contain an N-terminal double-glycine peptidase domain (SunT) (PDB:3K8U) |
| GC_00005385 | V | Defense mechanisms | | Predicted restriction endonuclease |
| GC_00005678 | V | Defense mechanisms | | Lantibiotic modifying enzyme (LcnDR2) (PDB:5DZT) |
| GC_00004931 | --- | No COG assignment | | No COG assignment |
| GC_00004971 | --- | No COG assignment | | No COG assignment |
| GC_00004979 | --- | No COG assignment | | No COG assignment |
| GC_00005070 | --- | No COG assignment | | No COG assignment |
| GC_00005072 | --- | No COG assignment | | No COG assignment |
| GC_00005090 | --- | No COG assignment | | No COG assignment |
| GC_00005213 | --- | No COG assignment | | No COG assignment |
| GC_00005242 | --- | No COG assignment | | No COG assignment |
| GC_00005278 | --- | No COG assignment | | No COG assignment |
| GC_00005306 | --- | No COG assignment | | No COG assignment |
| GC_00005345 | --- | No COG assignment | | No COG assignment |
| GC_00005470 | --- | No COG assignment | | No COG assignment |
| GC_00005486 | --- | No COG assignment | | No COG assignment |
| GC_00005492 | --- | No COG assignment | | No COG assignment |
| GC_00005533 | --- | No COG assignment | | No COG assignment |
| GC_00005624 | --- | No COG assignment | | No COG assignment |
| GC_00005632 | --- | No COG assignment | | No COG assignment |
| GC_00005656 | --- | No COG assignment | | No COG assignment |
| GC_00005658 | --- | No COG assignment | | No COG assignment |
| GC_00005667 | --- | No COG assignment | | No COG assignment |
| GC_00005672 | --- | No COG assignment | | No COG assignment |

Note: "No COG assignment" indicates that the best sequence alignment failed to meet the significance threshold, having an E-value >1×10^−5^.

**Table S5.** MALDI-TOF-MS detection of lipopeptides purified from acid precipitation.

| Product and observed mass peaks (m/z) | Assignment |
| --- | --- |
| Bacillomycin D | |
| 1031.5, 1053.5 | C14-Bacillomycin D [M + H, Na] |
| 1045.5, 1067.5 | C15-Bacillomycin D [M + H, Na] |
| Fengycin | |
| 1449.9, 1471.9 | C15-Fengycin A [M + H, Na] |
| 1463.9, 1485.9 | C16-Fengycin A [M + H, Na] |
| 1477.9, 1499.9 | C17-Fengycin A [M + H, Na] |
| 1477.9, 1499.9 | C15-Fengycin B [M + H, Na] |
| 1491.9, 1513.9 | C16-Fengycin B [M + H, Na] |
| 1505.9, 1527.9 | C17-Fengycin B [M + H, Na] |


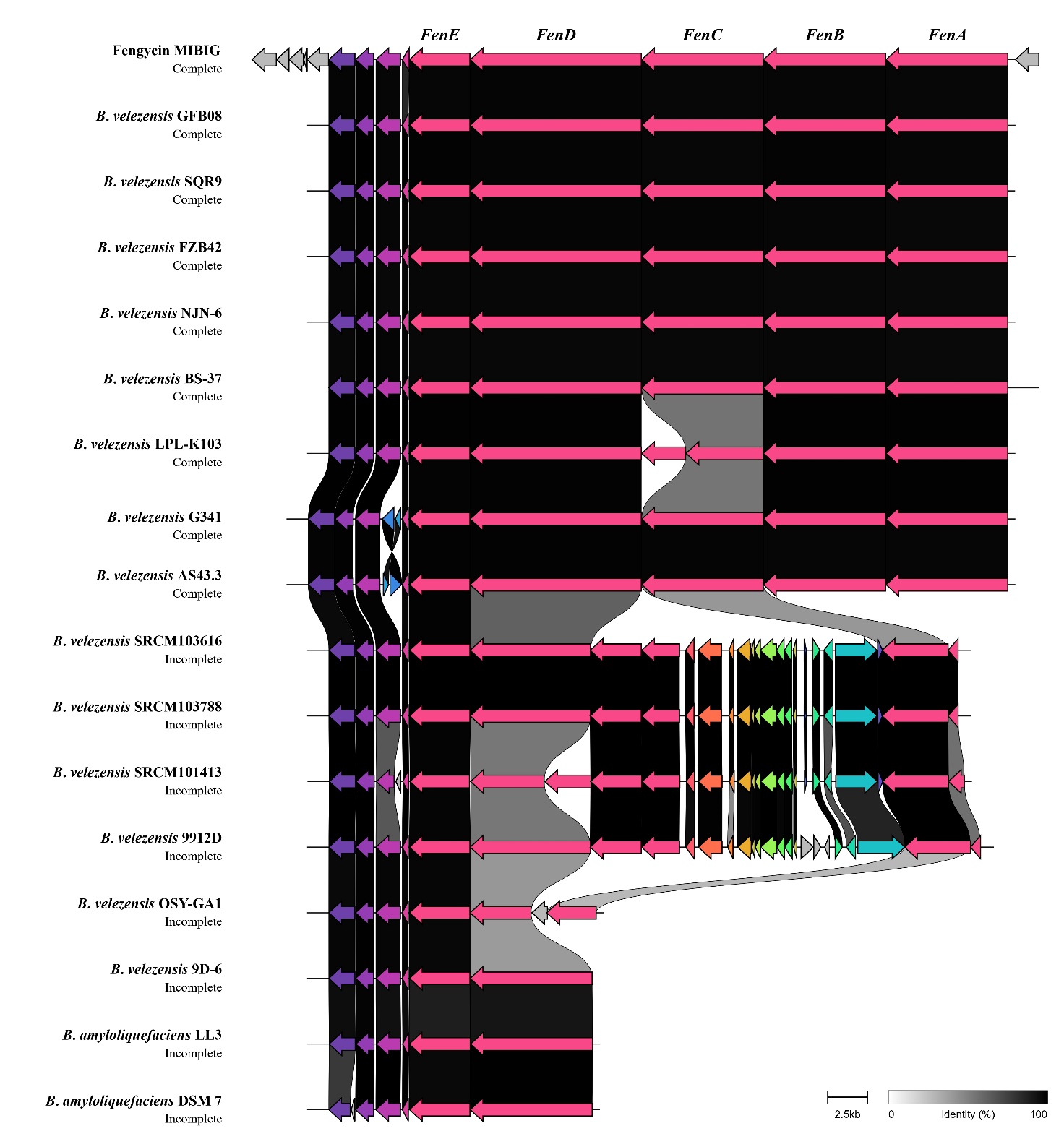
 **Fig. S1.** Comparison of biosynthetic gene clusters (BGCs) of fengycin family.


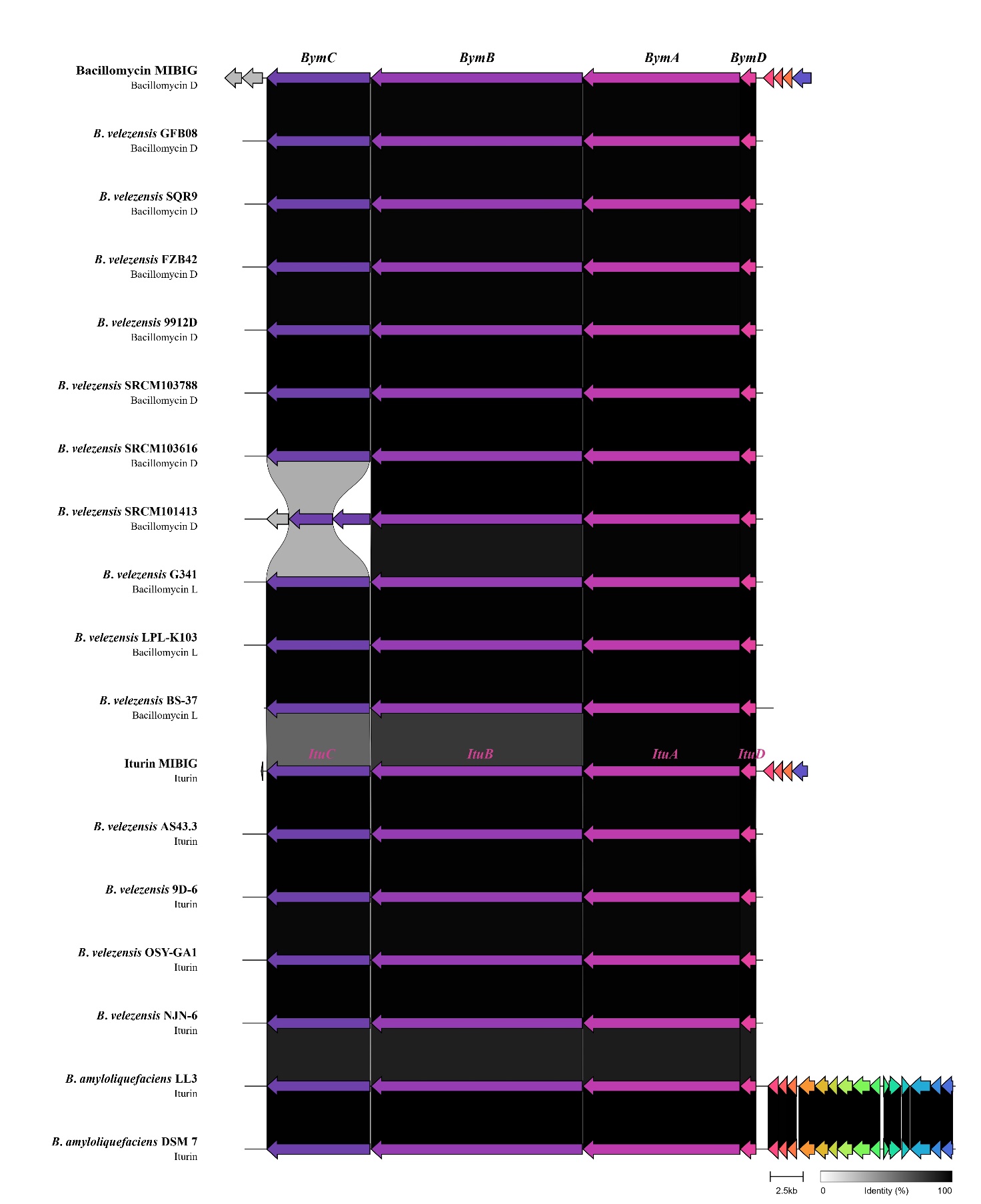


**Fig. S2.** Comparison of biosynthetic gene clusters (BGCs) of bacillomycin/iturin family.


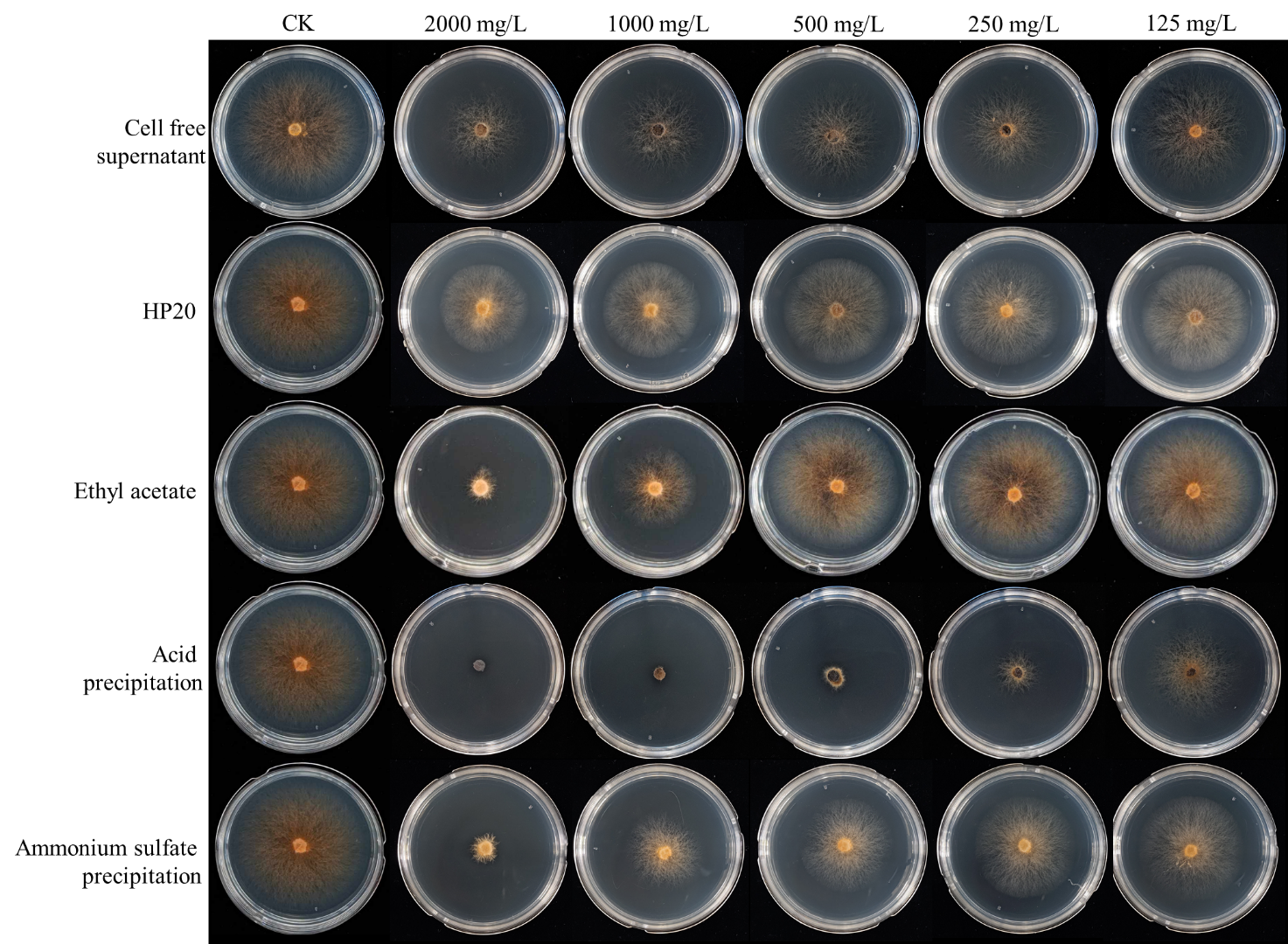


**A**

**B**


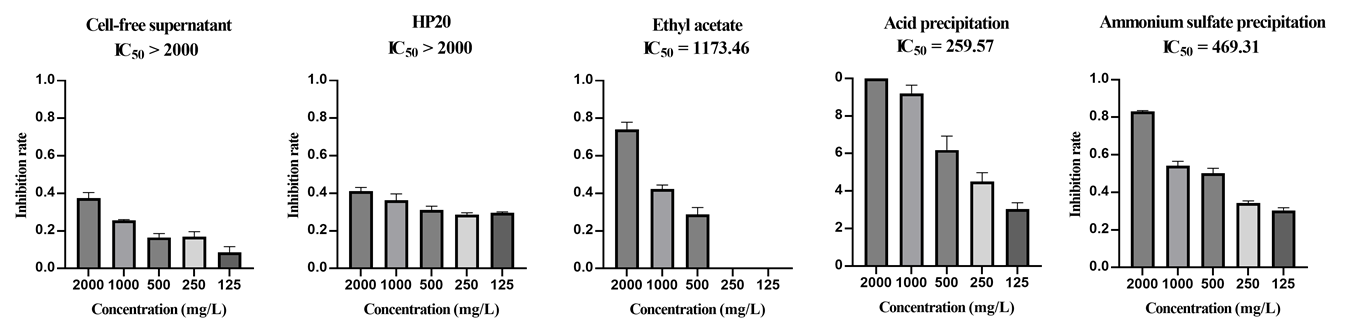


**Fig. S3.** Bioactivity assay of cell free supernatant and crude extracts from *Bacillus velezensis* GFB08 against *Colletotrichum spaethianum*. (A) Representative photographs showing the effects of the cell-free supernatant and crude extracts on mycelial growth. (B) Bar chart quantifying the percentage of mycelial growth inhibition.


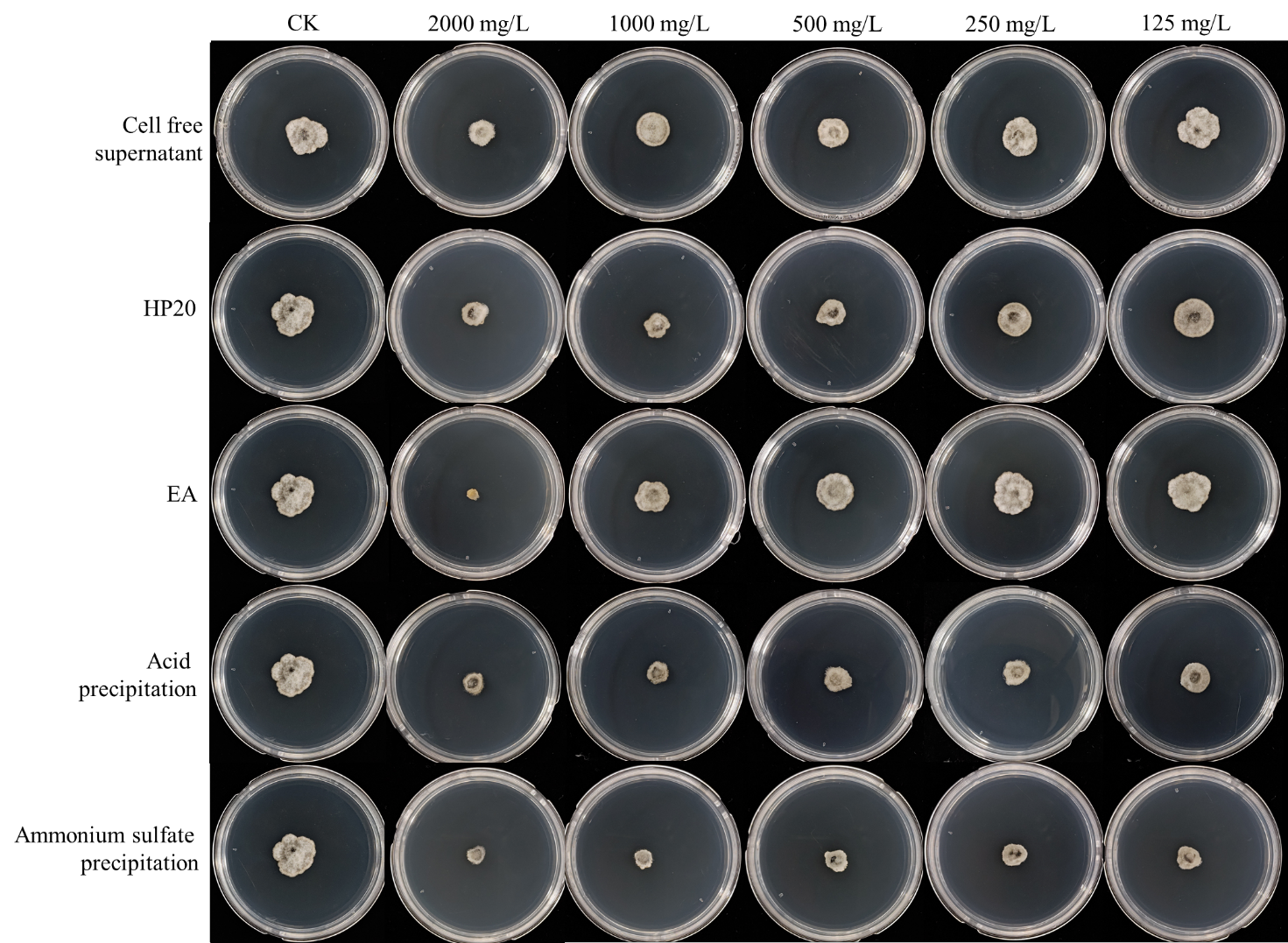


**A**

**B**


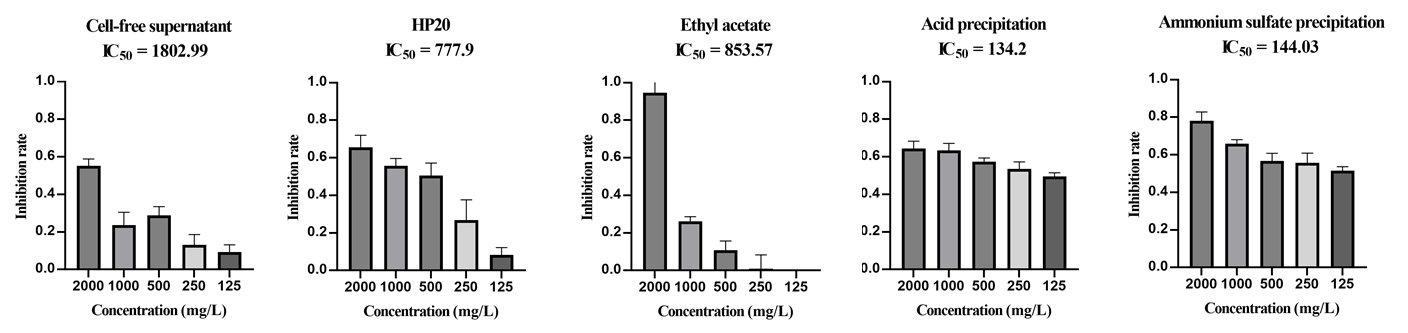


**Fig. S4.** Bioactivity assay of cell free supernatant and crude extracts from *Bacillus velezensis* GFB08 against *Colletotrichum circinans*. (A) Representative photographs showing the effects of the cell-free supernatant and crude extracts on mycelial growth. (B) Bar chart quantifying the percentage of mycelial growth inhibition.


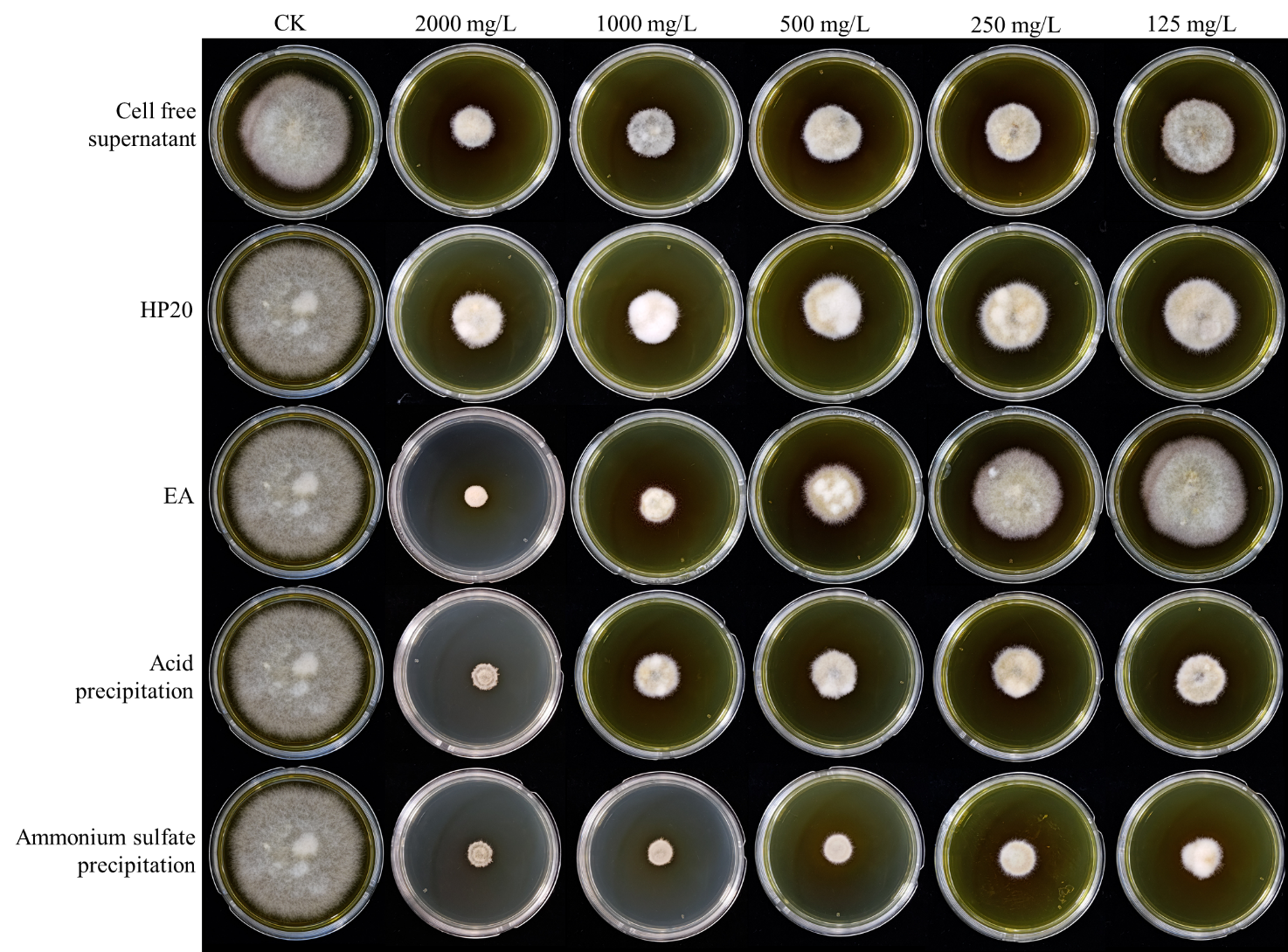


**A**

**B**


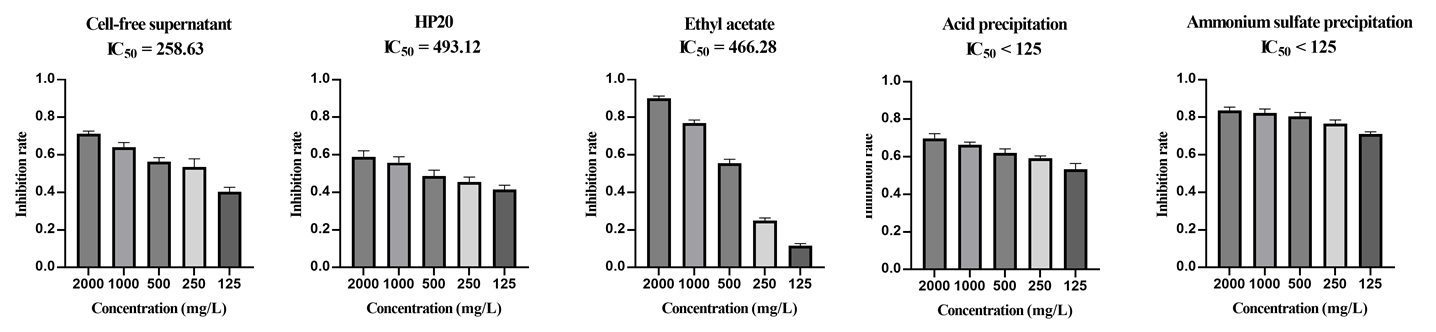


**Fig. S5.** Bioactivity assay of cell free supernatant and crude extracts from *Bacillus velezensis* GFB08 against *Stemphylium vesicarium*. (A) Representative photographs showing the effects of the cell-free supernatant and crude extracts on mycelial growth. (B) Bar chart quantifying the percentage of mycelial growth inhibition.

**
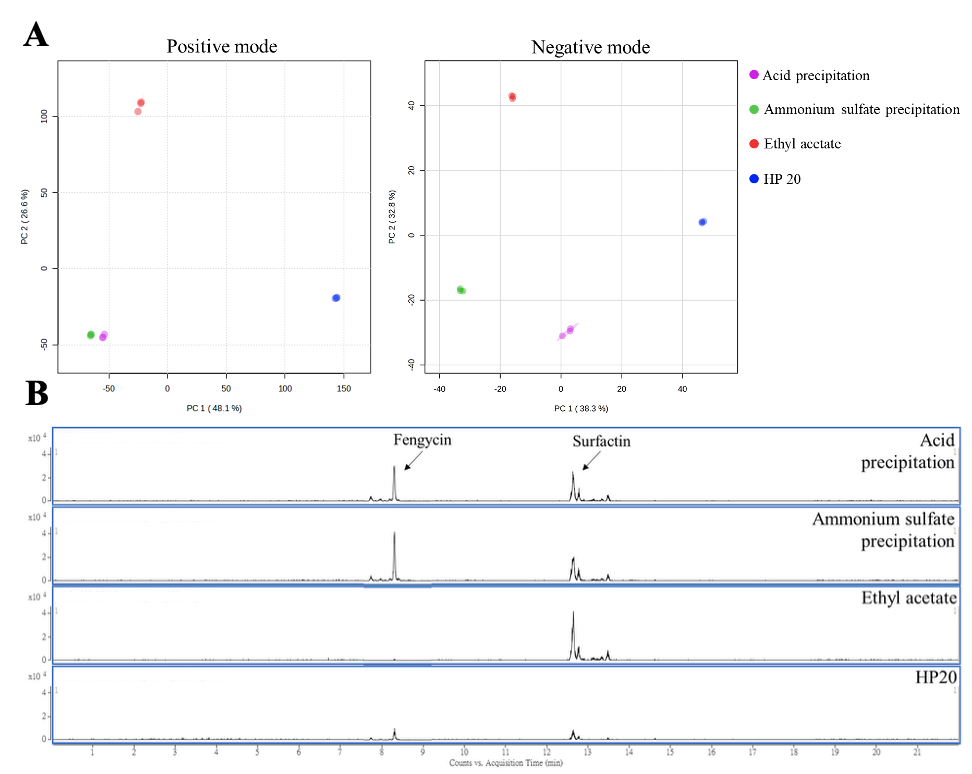
**

**Fig. S6.** LC-MS analysis of the metabolites in four extractions. (A) Principal components analysis (PCA) of metabolite profiles, (B) Targeted search of extracted ion chromatograms for surfactin and fengycin.

**
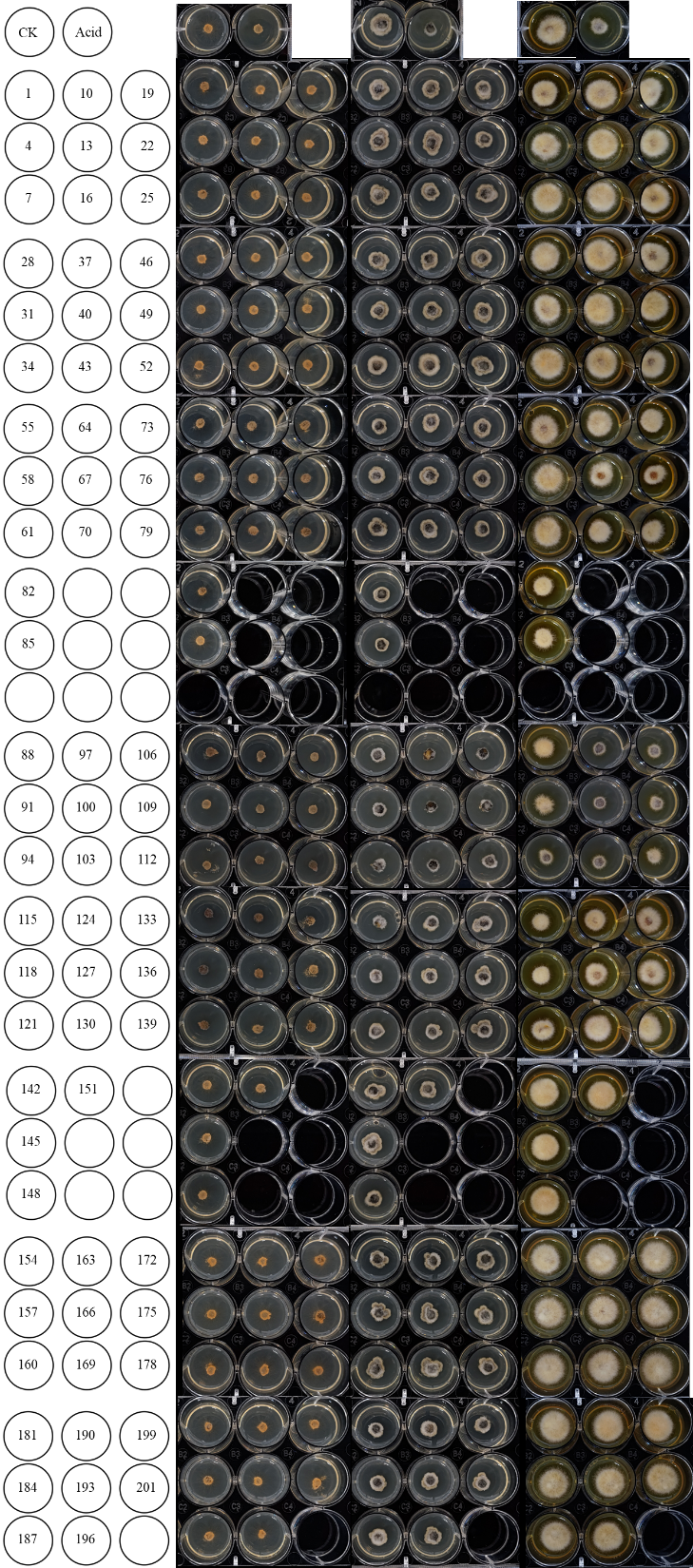
Fig. S7.** Antifungal bioactivity screening of selected fractions from Sephadex LH-20 column chromatography against Welsh onion foliar pathogens. Every third fractions were tested. Panels (left to right) display results against *C*. *spaethianum*, *C*. *circinans*, and *S*. *vesicarium*, respectively. Acid: 500 mg/L acid precipitation crude extracts


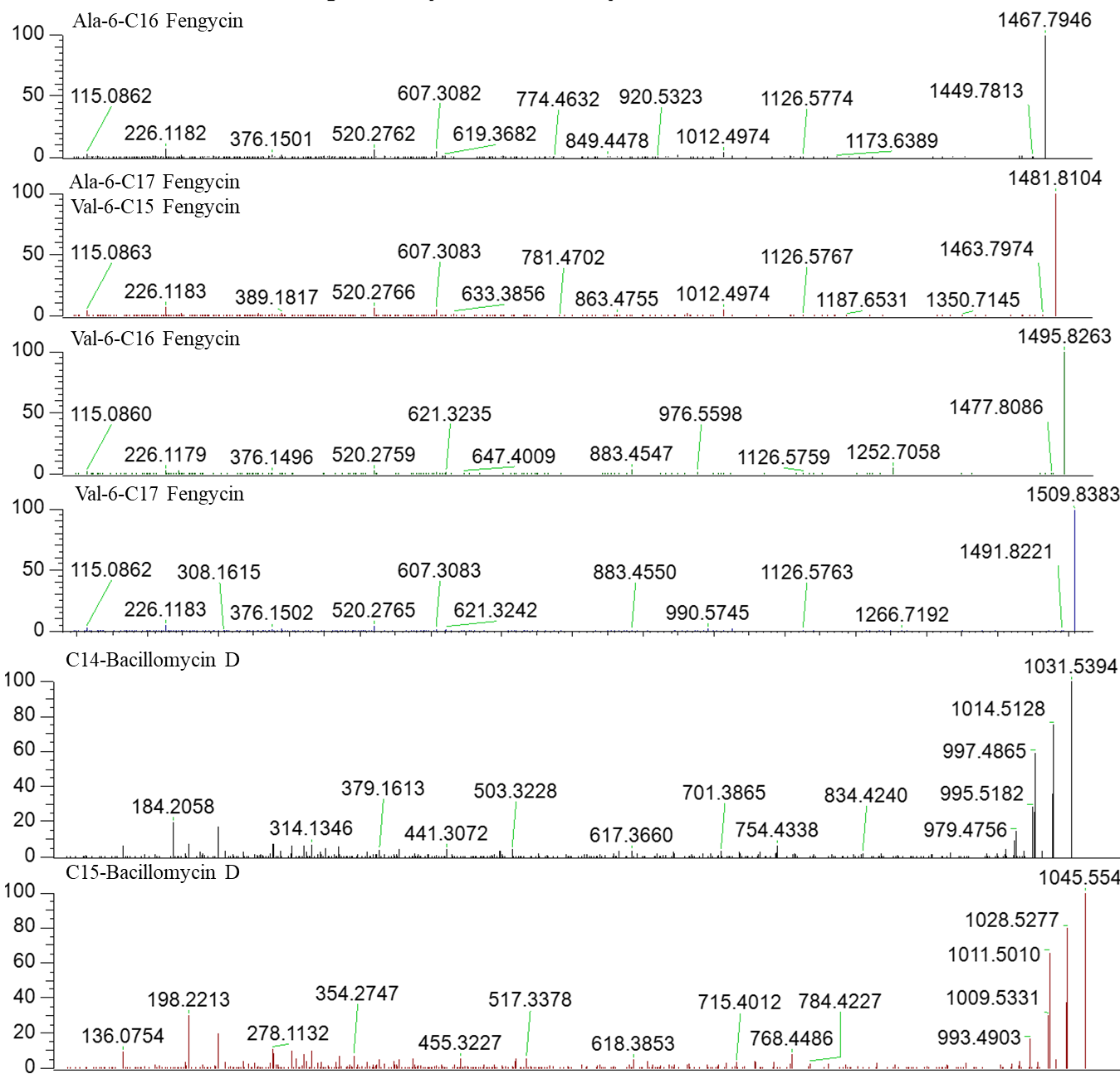


**Fig. S8.** MS/MS of fengycin and bacillomycin D isoforms from LC-MS analysis of fraction fengycin and bacillomycin D.

**
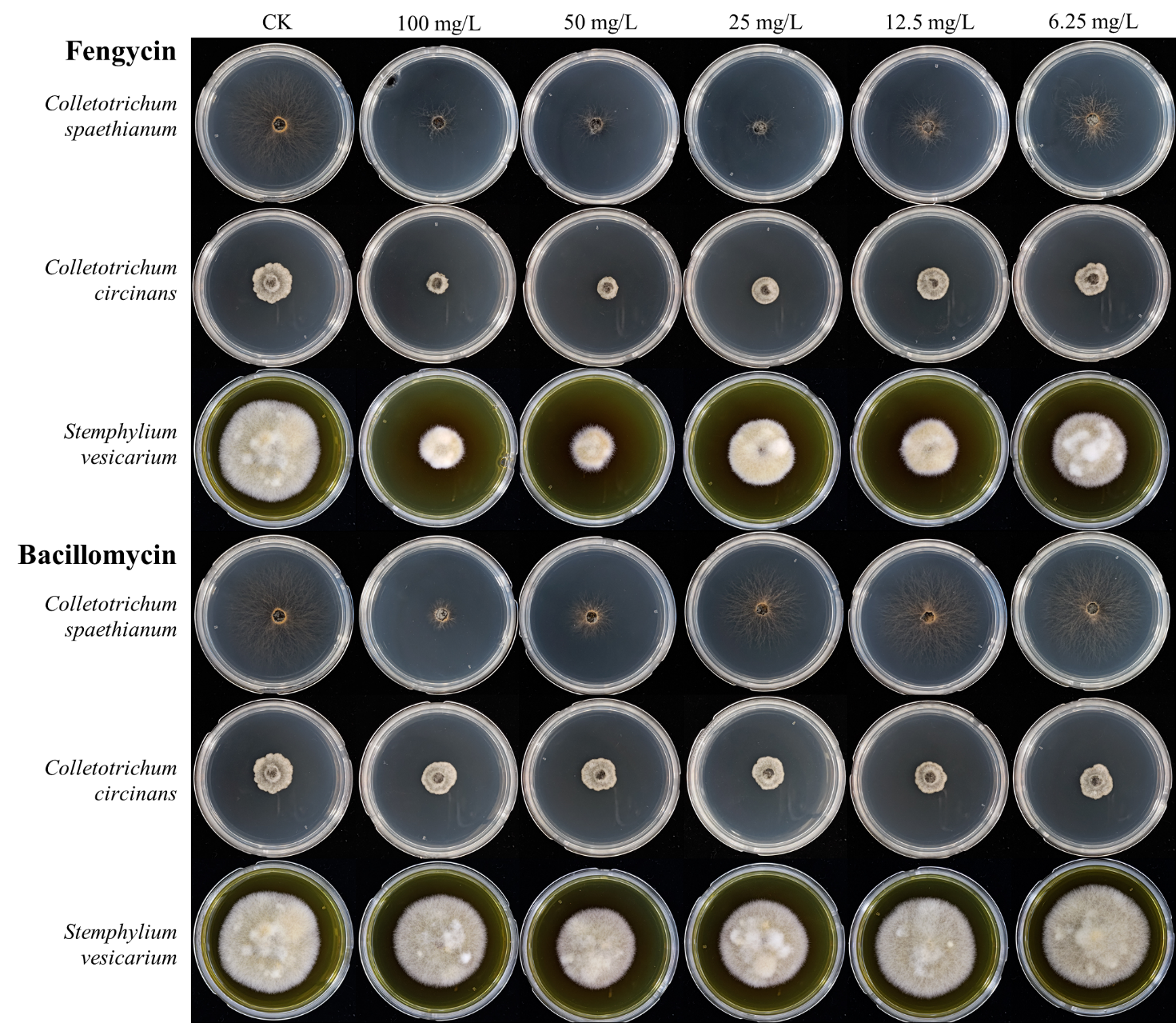
**

**Fig. S9.** Bioactivity assay of purified fengycin and bacillomycin D from *Bacillus velezensis* GFB08 against Welsh onion foliar pathogens.

**
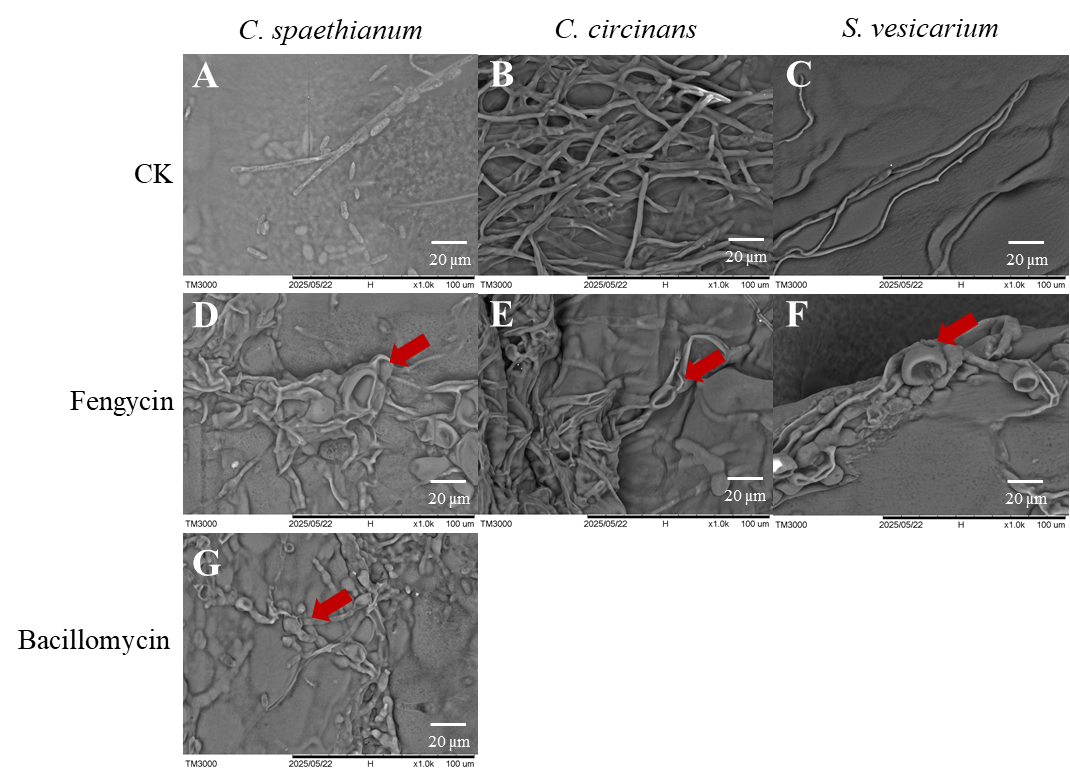
**

**Fig. S10.** Morphological changes of *Colletotrichum spaethianum*, *C*. *circinans*, and *S*. *vesicarium* hyphae treated with fengycin (100 mg/L) and bacillomycin D (100 mg/L) under scanning electron microscopy (SEM). Red arrow showed irregular expansion and rupture of hyphae treated with fengycin or bacillomycin. A to C: CK; D-F: Fengycin treatment; G: Bacillomycin D treatment.


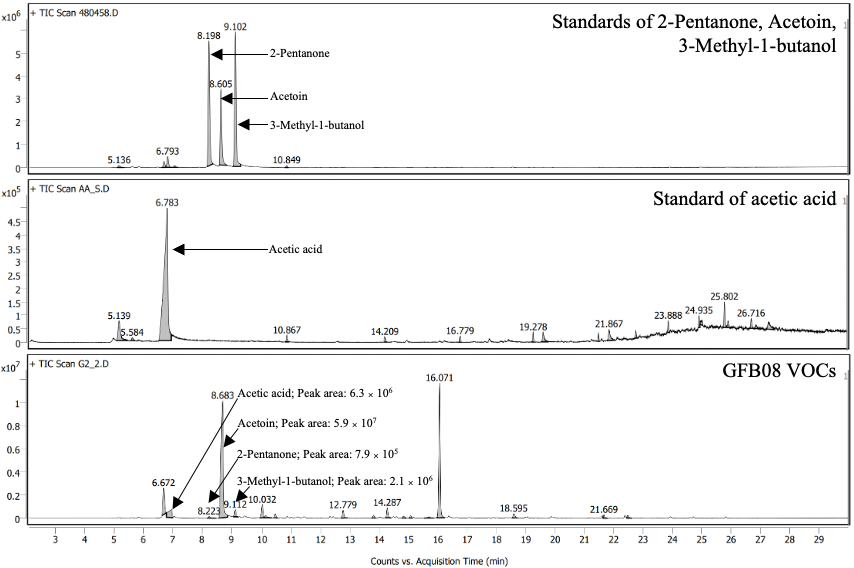


**Fig. S11.** GC-MS validation of four commercially purchased VOC standards.


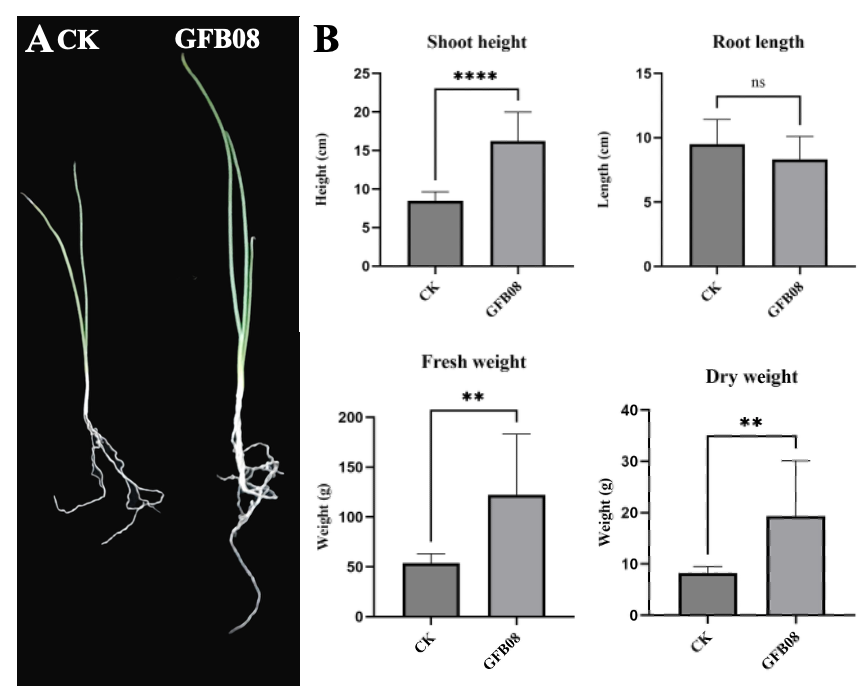


**Fig. S12.** Evaluation of plant growth-promoting ability of *Bacillus velezensis* GFB08 through seed soaking assay. (A) Representative photograph showing plant growth-promoting ability of *Bacillus velezensis* GFB08. (B) Bar chart quantifying the plant growth parameters. GFB08: *B*. *velezensis* GFB08; ns: not significant (p-value > 0.05); **: p-value < 0.01; ****: p-value < 0.0001.


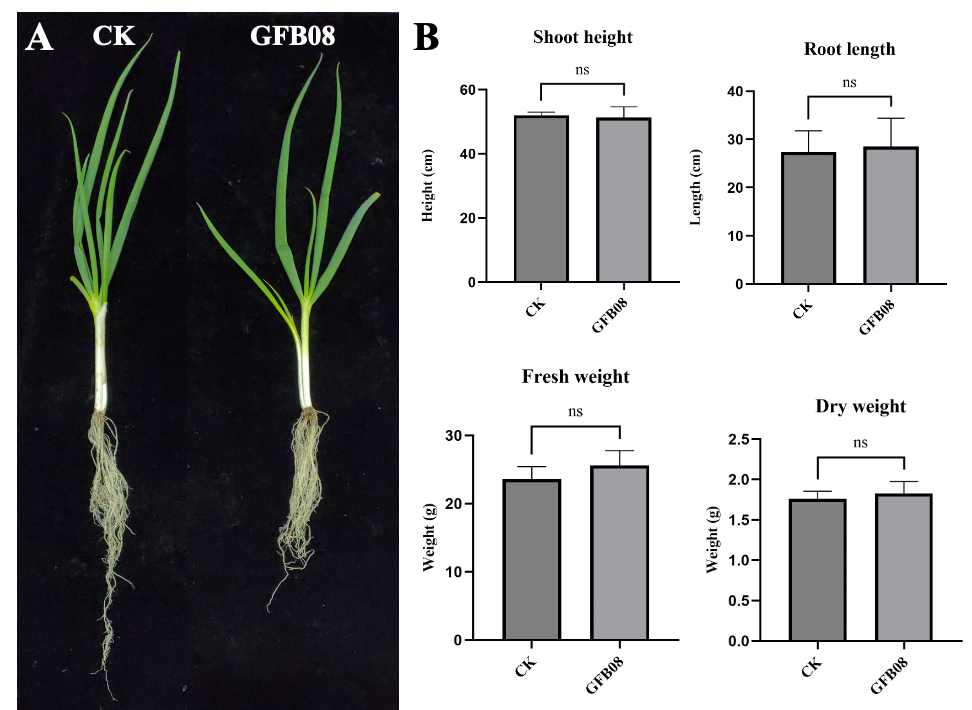


**Fig. S13.** Evaluation of plant growth-promoting ability of *Bacillus velezensis* GFB08 on Welsh onion up to four leave stage. (A) Representative photograph showing plant growth-promoting ability of *Bacillus velezensis* GFB08*.* (B) Bar chart quantifying the plant growth parameters. ns: not significant (p-value > 0.05).
